# Evaluation of *Fusobacterium nucleatum* Enoyl-ACP Reductase (FabK) as a Narrow-Spectrum Drug Target

**DOI:** 10.1021/acsinfecdis.3c00710

**Published:** 2024-04-10

**Authors:** Jacob T. Rutherford, Kristiana Avad, Chetna Dureja, Krissada Norseeda, Bibek GC, Chenggang Wu, Dianqing Sun, Kirk E. Hevener, Julian G. Hurdle

**Affiliations:** † Center for Infectious and Inflammatory Diseases, Institute of Biosciences and Technology, Department of Translational Medical Sciences, Texas A&M Health Science Center, Houston, Texas 77030, United States; ‡ Department of Pharmaceutical Sciences, College of Pharmacy, University of Tennessee Health Science Center, Memphis, Tennessee 38163, United States; § Department of Pharmaceutical Sciences, The Daniel K. Inouye College of Pharmacy, University of Hawaii at Hilo, Hilo, Hawaii 96720, United States; ∥ Department of Microbiology & Molecular Genetics, McGovern Medical School, University of Texas Health Science Center at Houston, Houston, Texas 77030, United States

**Keywords:** *F. nucleatum* pathogen, narrow-spectrum antimicrobial, gene silencing, fatty acid biosynthesis

## Abstract

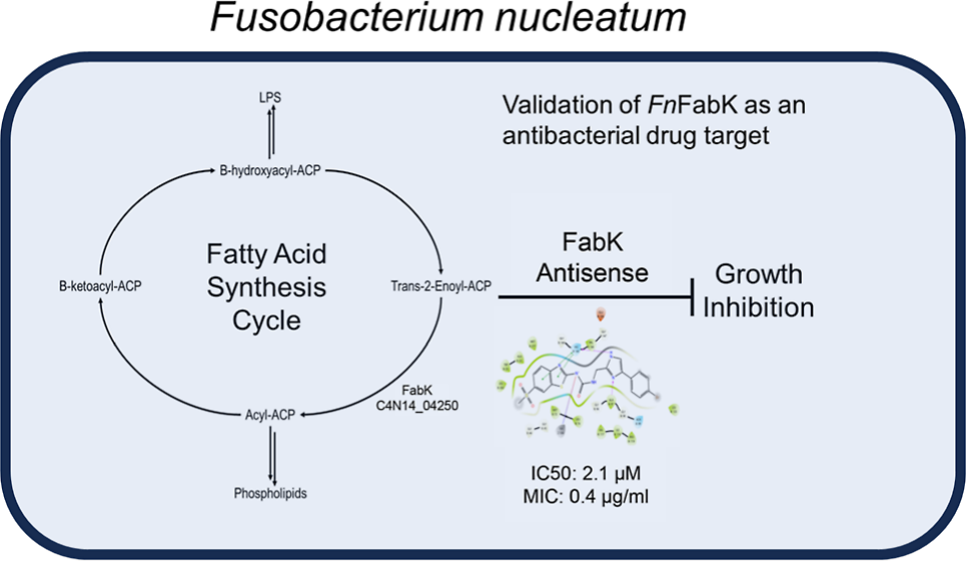

*Fusobacterium nucleatum*, a pathobiont inhabiting the oral cavity, contributes to opportunistic diseases, such as periodontal diseases and gastrointestinal cancers, which involve microbiota imbalance. Broad-spectrum antimicrobial agents, while effective against *F. nucleatum* infections, can exacerbate dysbiosis. This necessitates the discovery of more targeted narrow-spectrum antimicrobial agents. We therefore investigated the potential for the fusobacterial enoyl-ACP reductase II (ENR II) isoenzyme *Fn*FabK (C4N14_ 04250) as a narrow-spectrum drug target. ENRs catalyze the rate-limiting step in the bacterial fatty acid synthesis pathway. Bioinformatics revealed that of the four distinct bacterial ENR isoforms, *F. nucleatum* specifically encodes *Fn*FabK. Genetic studies revealed that *fabK* was indispensable for *F. nucleatum* growth, as the gene could not be deleted, and silencing of its mRNA inhibited growth under the test conditions. Remarkably, exogenous fatty acids failed to rescue growth inhibition caused by the silencing of *fabK*. Screening of synthetic phenylimidazole analogues of a known FabK inhibitor identified an inhibitor (i.e., 681) of *Fn*FabK enzymatic activity and *F. nucleatum* growth, with an IC_50_ of 2.1 μM (1.0 μg/mL) and a MIC of 0.4 μg/mL, respectively. Exogenous fatty acids did not attenuate the activity of 681 against *F. nucleatum*. Furthermore, *Fn*FabK was confirmed as the intracellular target of 681 based on the overexpression of *Fn*FabK shifting MICs and 681-resistant mutants having amino acid substitutions in *Fn*FabK or mutations in other genetic loci affecting fatty acid biosynthesis. 681 had minimal activity against a range of commensal flora, and it was less active against streptococci in physiologic fatty acids. Taken together, *Fn*FabK is an essential enzyme that is amenable to drug targeting for the discovery and development of narrow-spectrum antimicrobial agents.

## Introduction

**Figure 1:**
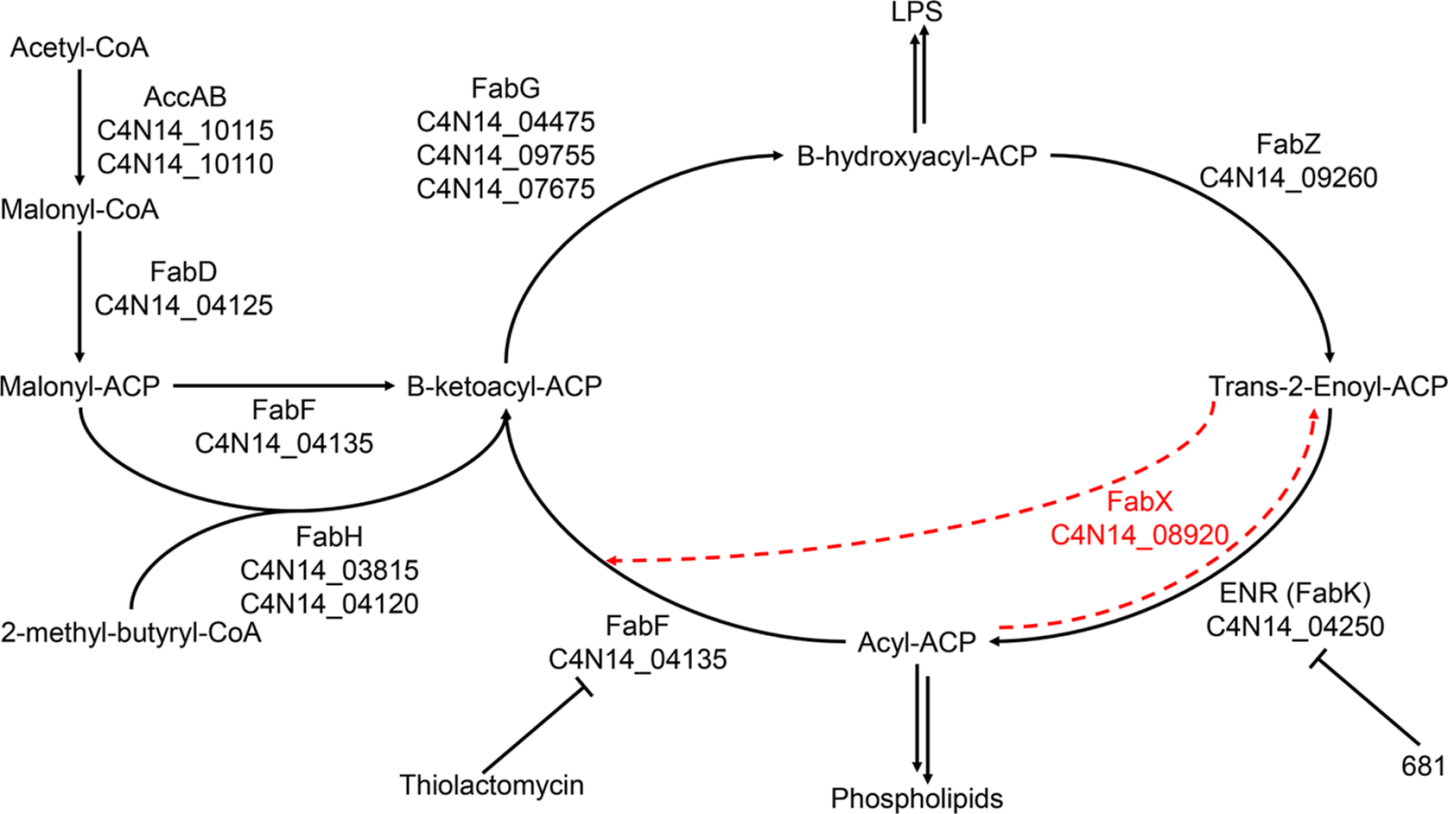
Overview of fatty acid synthesis in *F. nucleatum*. The initiation and elongation cycles of FAS-II are annotated with the genes encoding FAS-II proteins from *F. nucleatum* strain ATCC 23726. Highlighted in red is FabX, which is homologous to C4N14_08920; FabX is thought to synthesize unsaturated fatty acids. Thiolactomycin is a commercially available FabF inhibitor, while 681 is an analogue of a known phenylimidazole FabK inhibitor. The corresponding enzyme names are given in Table S1.

The Gram-negative oral pathobiont *Fusobacterium nucleatum* is implicated in multiple diseases, including periodontal disease, preterm births, inflammatory bowel disease, colorectal (CRC), and breast cancers.^1−5^ In periodontal disease, *F. nucleatum* assists periodontal pathogens such as *Porphyromonas gingivalis* by promoting their integration into the oral microbiota community. In the context of CRC, genomic analysis of patient biopsies has shown a frequent association of *F. nucleatum* with tumor tissues, as opposed to normal tissues.^6,7^*F. nucleatum* expresses virulence factors that contribute to the development of a pro-tumorigenic environment, which includes suppression of the immune system,^8,9^ promotion of chemoresistance,^10^ and induction of inflammation.^4,11^ The discovery that *F. nucleatum* may promote the progression and metastasis of cancers has spurred research aimed at identifying antivirulence and antimicrobial agents to eradicate *F. nucleatum* from tumor environments.^12−16^ The advantage of eliminating *F. nucleatum* was demonstrated in a study by Bullman et al.,^12^ wherein mice treated with the antimicrobial metronidazole exhibited a decrease in *F. nucleatum* positive xenograft CRC tumors. Highlighting the importance of this work, there are two ongoing clinical trials exploring the utility of metronidazole as an adjuvant to standard of care approaches for CRC.^17,18^ However, metronidazole has limitations as it is a broad-spectrum antianaerobic antibiotic that is likely to promote dysbiosis during the treatment of CRC. Thus, there is a need for narrow-spectrum antimicrobial agents that target *F. nucleatum* while minimizing collateral damage of the normal microbiota. Recent research has identified different potential strategies, including aspirin, that kill *F. nucleatum* and shows efficacy in Apc^Min/+^ mice;^13^ bacteriophages that target *F. nucleatum*;^19−22^ and derivatives of the natural product higenamine that inhibits *F. nucleatum* growth.^23^ To address the need for *F. nucleatum*-specific antimicrobial agents, we reviewed prior literature^24,25^ on antimicrobial drug targets with the view of identifying large-scale differences between *F. nucleatum* and common gut flora (i.e., Bacteroides and Firmicutes).^26^ This led us to explore enoyl-ACP reductase (ENR; Figure 1), which catalyzes the rate-limiting step of the bacterial type II fatty acid synthesis pathway (FAS-II) since these enzymes are attractive targets for narrow-spectrum drugs.^27^ FAS-II is required for bacteria to synthesize their phospholipid membranes. Additionally, Gram-negative bacteria like *F. nucleatum* depend on FAS-II to synthesize β-hydroxyacyl-ACPs, which are essential precursors for the lipid component of lipopolysaccharide outer membranes.^28,29^ In contrast, humans adopt the type I fatty acid synthesis pathway that is conducted by a large multifunctional enzyme.^30^ Among bacteria, there are primarily four ENR isoenzymes, of which FabI, FabL, and FabV are structurally related short-chain dehydrogenases that use NADPH in their enoyl reduction reactions.^31^ Conversely, the fourth isoform, FabK, is a flavoenzyme that uses FMN and NADPH.^32^ The occurrence of four isoenzymes makes ENRs attractive targets for antimicrobial agents with a narrower spectrum of action. The feasibility of this concept is exemplified by afabicin, a selective inhibitor for FabI that is in clinical development for *Staphylococcus aureus* skin and skin structure infections, and isoniazid that inhibits the mycobacterial ENR (InhA) and is an antituberculosis drug.^33,34^

Our bioinformatic analysis of *F. nucleatum* genomes revealed that they encode FabK as their only ENR (Figure 1). We, and others, have reported that FabK enzymes can be selectively inhibited by small molecules, with the phenylimidazole class of compounds representing potential drug candidates. Studies of a lead phenylimidazole, designated as 296, inhibited *Clostridioides difficile* FabK in cellular and enzymatic assays and was efficacious in treating *C. difficile* infection (CDI) in mice.^26,35,36^ Importantly, 296 did not appear to significantly disrupt the gut microbiome of mice, in contrast to the standard-of-care drugs for CDI, vancomycin, and fidaxomicin.^35^ The FabK proteins from *F. nucleatum* (*Fn*FabK) and *C. difficile* (*Cd*FabK) are closely related, with 56% overall identity and 74% overall similarity, and their active sites differ by only a single residue at amino acid position 46; Figure S1. This prompted us to investigate whether *Fn*FabK could be a target. In this study, we report that *Fn*FabK is a druggable antimicrobial target that is essential for the growth of *F. nucleatum*.

## Results and Discussion

### Bioinformatic Analysis

*The F. nucleatum* FAS-II pathway was assembled from DELTA-BLAST searches against the genome of strain 23726 (Figure 1; Table S1; accession no. SAMN00001497). Given the phylogenetic relatedness of *F. nucleatum* to Gram-positive firmicutes, we adopted enzymes from the FAS-II cycle of *Enterococcus faecalis* FA2–2 (accession no. SAMN22569088) to identify *F. nucleatum* FAS-II enzymes, using criteria of >80% query coverage and >35% identity. Enzymes from *E. faecalis* were used due to the extensive literature available on the *E. faecalis* FAS-II pathway.^37−40^ We identified homologues for each initiation and elongation step of FAS-II. Crucially, *Fn*FabK was the only ENR in strain 23726 and all other annotated *F. nucleatum* genomes (*n* = 51) studied from GenBank. No homologues of FabI, FabL, or FabV were found. All *Fn*FabK contained the distinctive FMN binding motif that is absent in other ENR isoenzymes and had >90% sequence identity. Hence, we further studied *Fn*FabK, considering that the homologous *Cd*FabK is a drug target in *C. difficile*.^26^

### Genetic Essentiality of *Fn*FabK

The genetic essentiality of *Fn*FabK was examined through gene deletion trials and gene silencing. Initially, we attempted to delete *fabK* by allelic exchange using a galactose kinase (GalK)-based genetic system in *F. nucleatum* 23726Δ*galK* (a *galK-*deletion mutant).^41^ The suicide vector containing the *fabK* allelic cassette was integrated into the chromosome adjacent to *fabK*, and cells were treated with 2-deoxy-d-galactose to counter-select and identify the second crossover mutants as those with potential *fabK* deletions. However, PCR analysis of colonies from two independent experiments (*n* = 10 each) showed that they all carried wild-type *fabK*. This indicated that *fabK* was not disrupted, and it was likely to be indispensable for *F. nucleatum* growth.

**Figure 2:**
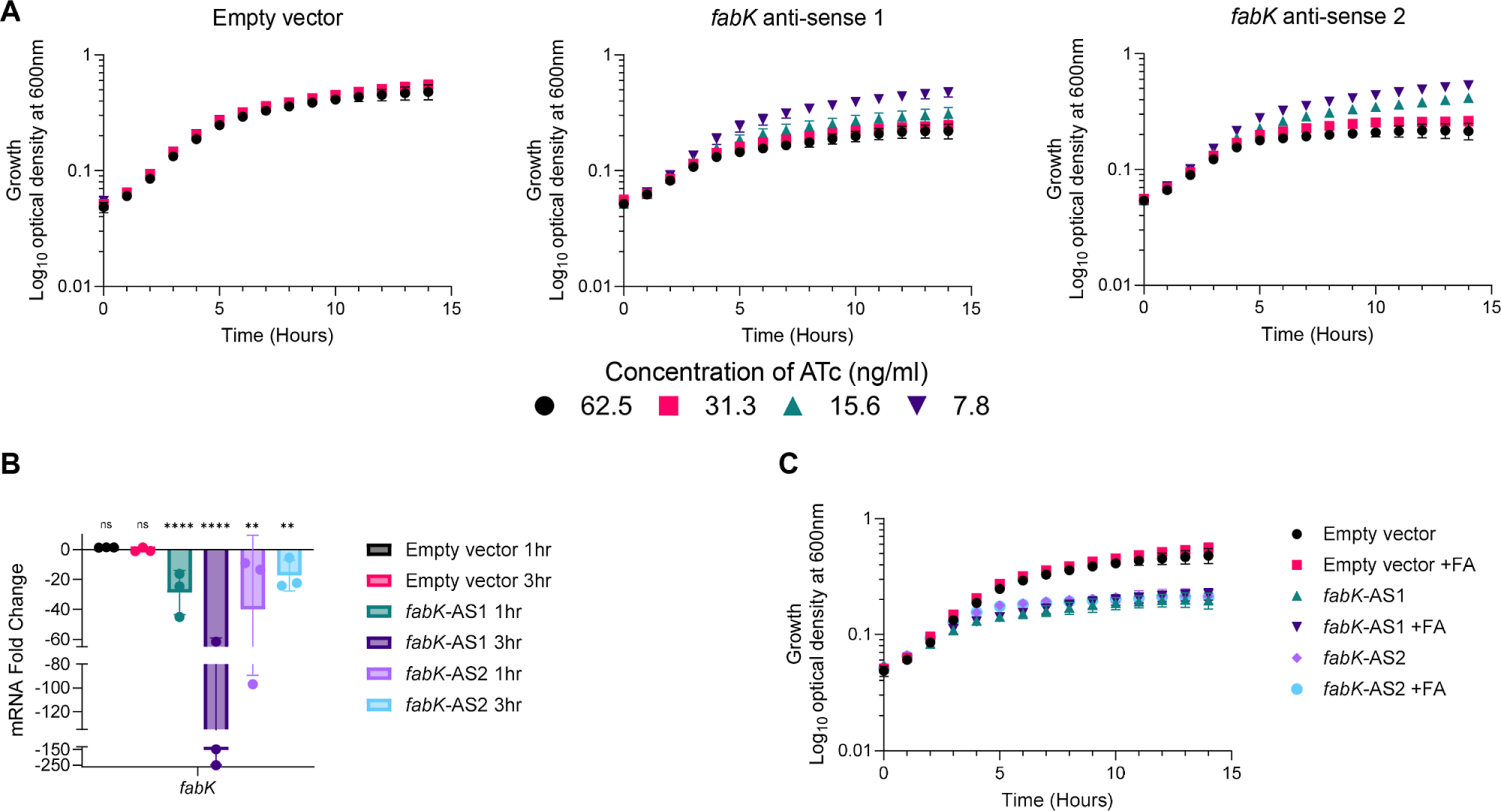
Gene silencing of *F. nucleatumfabK* inhibits growth. (A) Antisenses FabKAS1 and FabKAS2 were cloned into *F. nucleatum* 23726 and induced with indicated concentrations of anhydrotetracycline (ATc) to evaluate effects on growth compared with the empty vector control. Growth was inhibited in an ATc concentration-dependent manner; data are plotted as the mean of three biological replicates; error bars indicate the SEM. (B) RT-qPCR confirmed that FabKAS1 and FabKAS2 blocked the mRNA site targeted by the antisenses. After ATc induction or ethanol (EtOH) control treatment for 1 and 3 h, RNA was collected for RT-qPCR. The data was normalized to the 16S rRNA and fold-change is relative to the uninduced (EtOH) corresponding strain. Each data point is from three biological replicates, with each having two technical replicates, error bars indicate the standard deviation. Statistical analysis was done using a two-way ANOVA on the dCT values, comparing induced to uninduced for each strain and time point. ns, *p* > 0.05; **, *p* < 0.01; ****, *p* < 0.0001. (C) Supplementation with a mixture containing different fatty acids (FAs) did not reinstate growth when the antisenses were induced with ATc (62.5 ng/mL); the FAs were myristic acid (C14), palmitic acid (C16), stearic acid (C18), oleic acid (C18:1Δ9), linoleic acid (C18:2Δ9,12), and linolenic acid (C18:3Δ9,12,15); concentrations of FAs can be found in Table S2; data are plotted as the mean of three biological replicates, and error bars indicate the SEM.

Since our deletion approach utilized a genetically modified strain lacking *galK* and we did not attempt to delete the gene while the strain was complemented with a codon-altered *fabK*, we next employed gene silencing in the wild-type strain to confirm that *F. nucleatumfabK* is essential. Thus, we adopted gene silencing as a known target validation approach^24,42−44^ by adapting a paired-termini gene silencing method to inhibit *fabK* mRNA translation with antisense nucleotides that are directed toward the ribosome binding site.^26^ The *F. nucleatum* antisense vector (pHFK2) was constructed by cloning the paired-termini and anhydrotetracycline (ATc)-inducible pTET promoter sequences from the Clostridial vector pMSPT into the *F. nucleatum* cloning vector pCWU6 (Figure S2). Next, two different antisense nucleotides (100 bp) were cloned into pHFK2, i.e., specifically, FabK antisense 1 (FabKAS1) targeted the 50 bp upstream and downstream regions of the *fabK* start codon, whereas FabK antisense 2 (FabKAS2) targeted 25 bp upstream and 75 bp downstream of the start codon. Following induction with ATc, both FabKAS1 and FabKAS2 arrested the growth of *F. nucleatum* 23726 in an ATc concentration-dependent manner (Figure 2A). RT-qPCR confirmed that gene silencing inhibited the *fabK* mRNA ribosome binding region, as significantly fewer transcripts were detected for the antisense regions (Figure 2B). Interestingly, exogenous fatty acids could not relieve *F. nucleatum* growth inhibition caused by the antisenses (Figure 2C), indicating that *fabK* remained essential even when *F. nucleatum* cultures were provided with host lipids. We adopted a mixture of fatty acids of varying chain lengths and saturation (described in the Materials and Methods; Table S2) that are based on the composition of free fatty acids in the oral cavity^45^ and 0.1% v/v of Tween-80 (as a source of oleic acid, C18:1 *cis*-9; Figure S3). Thus, under these test conditions, *fabK* appears to be indispensable for *F. nucleatum* growth irrespective of the presence of exogenous fatty acids. Nonetheless, further genetic experiments may be warranted to determine whether *F. nucleatumfabK* is essential under different conditions (e.g., gene deletion in strains complemented with codon-altered *fabK* cloned with or without a conditional promoter).

### Identification of a Small-Molecule Inhibitor of *Fn*FabK

**Figure 3:**
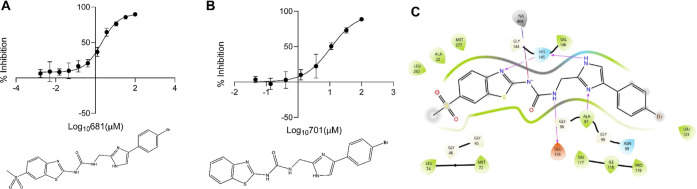
Identification of a small-molecule inhibitor of *Fn*FabK. (A,B) Compounds 681 (A) and 701 (B) were active against *Fn*FabK with IC_50_ 2.1 μM (95% CI 1.5 to 3.1 μM) and 10.64 μM (95% CI 6.6 to 32.3 μM), respectively, from three experimental repeats; these correspond to concentrations of 1.0 μg/mL (681; 95% CI 0.7 to 1.6 μg/mL) and 4.6 μg/mL (701; 95% CI 2.8 to 13.8 μg/mL). (C) model of compound 681 in the binding pocket of *Fn*FabK from *F. nucleatum* 23726. Arrows indicate the likely interactions of compound 681 with amino acids in *Fn*FabK; histidine-145 is the catalytic residue for *Fn*FabK.

To identify *Fn*FabK inhibitors with antimicrobial activity, we tested the susceptibility of *F. nucleatum* 23726 to a panel of *Cd*FabK inhibitors. The panel included phenylimidazole 296, a known FabK inhibitor, and 17 recently reported analogues of 296 (1-[4-(4-bromophenyl)-1*H*-imidazole-2-yl]methyl-3-[5-(pyridin-2-ylthio)thiazol-2-yl]urea).^36^ The test panel had reported IC_50_s of 0.1 to >10 μM against *Cd*FabK and MICs of 1.6 to >100 μg/mL against *C. difficile* cells.^36^ However, against *F. nucleatum* 23726, the MICs of compounds ranged from 0.4 to >100 μg/mL (Table 1). The most active compound (MIC = 0.4 μg/mL) was 681 (Figure 3A), a 6-MeSO_2_-benzothiazole derivative 1-((4-(4-bromophenyl)-1*H*-imidazole-2-yl)methyl)-3-(6-(methylsulfonyl)benzo[*d*]thiazol-2-yl)urea. Compound 681 stood out as the least lipophilic molecule, with a Log *D* (pH 7.4) value of 3.10, in contrast to 296, which had a Log *D* (pH 7.4) value of 4.52 and was inactive against *F. nucleatum* (MIC > 100 μg/mL). The other active compound was the benzothiazole derivative 701 (MIC = 1.6 μg/mL; Figure 3B), which resembled 681 but lacked the 6-MeSO_2_ substituent in the benzothiazole group; 701 had a Log *D* (pH 7.4) of 4.26. Since molecules that are more hydrophilic are generally more prone to have anti-Gram-negative activity,^46^ we initially speculated that the cellular activity of 681 might be partly due to its lower lipophilicity, which might facilitate penetration through the *F. nucleatum* outer membrane.

**Table 1:** Activity of Previously Reported *C. difficile* FabK Inhibitors against *F. nucleatum* 23726 and *E. faecalis* FA2-2 Strains; Results Are from Three Biological Replicates

	MIC (μg/mL)			
compound^a^	*Fn* 23726^b^	*Ef fabK*+^c^	*Ef fabI*+^d^	*Ef*wild-type^e^
296 (5a)	100	<0.1	100	100
701 (6a)	1.6	<0.1	100	100
702 (5d)	100	<0.1	100	100
703 (5i)	100	<0.1	100	100
670 (6i)	100	<0.1	100	100
671 (6g)	25	<0.1	50	50
673 (6d)	3.1	<0.1	6.3	6.3
674 (6j)	100	<0.1	100	100
675 (8b)	100	0.8	100	100
676 (8a)	100	0.2	100	100
679 (5b)	6.3	<0.1	100	100
680 (5h)	100	<0.1	100	100
681 (6f)	0.4	<0.1	100	100
682 (5e)	3.1	<0.1	100	100
683 (5g)	3.1	<0.1	100	100
684 (5f)	3.1	<0.1	100	100
689 (6h)	6.3	<0.1	25	25
690 (6k)	25	<0.1	25	25

a Shown in parentheses is the previously published nomenclature for the compounds.

b *F. nucleatum* (*Fn*) 23726.

c *E. faecalis* (*Ef*) *fabK*+ possesses only FabK.

d *Ef fabI*+ possesses only FabI.

e *Ef* wild-type possesses both FabI and FabK.

### 681 Inhibits *Fn*FabK and Directly Interacts with Key Residues in the Catalytic Site

Compounds 681 and 701 inhibited the enoyl reductase activity of purified *Fn*FabK in a dose-dependent manner, with mean IC_50_s of 2.1 μM (equivalent to 1.0 μg/mL; 681) and 10.6 μM (equivalent to 4.6 μg/mL; 701) from three replicates, respectively (Figure 3A,B). The Hill coefficient of 681 was 1.01 (with a 95% confidence interval of 0.70–1.48), which is close to the ideal value of 1.000 for the Hill slope. Similarly, the Hill coefficient of 701 was 1.06 (95% confidence interval of 0.59–1.75). These were comparable to previously reported inhibition of *Cd*FabK with a 681 IC_50_ of 0.45 μM (equivalent to 0.2 μg/mL; Hill slope 0.97) and a 701 IC_50_ of 1.88 μM (equivalent to 0.8 μg/mL; Hill slope 0.94).^36^ Interestingly, the fivefold higher IC_50_ of 701 compared to 681 roughly correlates with the fourfold difference in MIC between the two compounds. Hence, the difference in activity between 681 and 701 may likely be due to binding affinity rather than penetration into cells, as was initially speculated; however, drug penetration was not tested and was beyond the scope of the study.

Interactions of 681 with *Fn*FabK were examined using a homology model of the enzyme that was based on the reported crystal structure of *Streptococcus pneumoniae* FabK (Figure 3C).^47^*Fn*FabK and *Sp*FabK proteins are 56% identical with 71% similarity. Their active site residues are 94% identical, differing only in the presence of an ASN residue at the *Fn*FabK GLN-46 position. The docking pose of 681 closely matched the binding orientation of the phenylimidazole compound (an analogue of 296) that was experimentally determined in the *Sp*FabK cocrystal structure (PDB ID: 2Z6J).^47^ The phenylimidazole ring system of 681 occupied the same position in the *Fn*FabK active site as the homologous ring system of the 296 analogue in the *Sp*FabK structure. Similarly, the thiazole ring and urea groups of 681 engaged in a pi-stacking interaction with the FMN cofactor in a similar orientation as the 296-analogue in the *Sp*FabK structure. The methyl sulfone occupied the same region of the active site as the pyridine ring of the 296-analogue, potentially engaging in a hydrogen-bond interaction with ASN-46 (i.e., *F. nucleatum* numbering) in *Sp*FabK and *Cd*FabK enzymes. However, this interaction is not possible in *Fn*FabK since it carries a GLY-46 residue, which may explain why 681 has a more potent IC_50_ (0.45 μM) against *Cd*FabK.^36^

Compounds 681 and 701 differ only by the presence of a methyl-sulfone group at the 6-position of the benzothiazole ring system in 681. While this functional group does not appear to directly engage the *Fn*FabK active site in an electrostatic interaction, as discussed above, the electron-withdrawing character of the functional group likely improves 681 binding by lowering the p*K*_a_ of the urea nitrogen adjacent to the benzothiazole ring (p*K*_a_ ∼8), allowing the compound to coordinate an active site metal as an anion that is present in FabK enzymes. We previously discussed this structure–activity relationship (SAR) observation for inhibitors of *Cd*FabK^36^ and have noted this in our deposited *Cd*FabK cocrystal structure (PDB ID: 7L00), which carries an active site sodium. Further, the lipophilic character of the methyl sulfone of 681 may improve binding affinity as these inhibitors are fatty acid substrate mimics. These chemical properties likely explain the difference in biochemical activity noted between the two compounds (2.1 μM for 681 vs 10.6 μM for 701). Since 681 had stronger inhibition in both the enzymatic and the whole-cell MIC assays, it was selected for further characterization.

### *Fn*FabK Is an Intracellular Target of 681

We identified FabK as the intracellular target of 681 in three ways: first, by evaluating its activity against isogenic *E. faecalis* strains^39^ that harbor either FabI or FabK; second, by measuring the effect on 681 MICs when *Fn*FabK is expressed from the ATc-inducible pTET promoter in *F. nucleatum*; and third, by generating and analyzing 681-resistant *F. nucleatum* mutants. *E. faecalis* encodes both *fabI* and *fabK* and can survive when either ENR is deleted.^39^ To define the selectivity of 681, we used *E. faecalis* FA2–2 isogenic mutants that carried either FabI or FabK. Compound 681 was inactive (MIC > 100 μg/mL) against wild-type *E. faecalis* FA2–2 and the isogenic deletion mutant *E. faecalis* FA2–2Δ*fabK* that expressed only FabI. Conversely, 681 was potent against *E. faecalis* FA2–2Δ*fabI* that only encoded FabK (i.e., MIC = 0.005 μg/mL). Next, when FabK was overexpressed in wild-type *F. nucleatum* 23726, cells became totally resistant to 681 (MIC > 100 μg/mL) but remained susceptible to thiolactomycin (MIC = 3.9 μg/mL), a control compound that inhibits FabF/FabB (3-oxoacyl-[acyl-carrier-protein] synthase 2/3-oxoacyl-[acyl-carrier-protein] synthase 1) (Table 2).^48^ These observations strongly suggest that within a cellular context 681 displayed selectivity for FabK.

**Table 2:** Activities of 681 and Thiolactomycin (TLM) against *F. nucleatum* 23726 Transformed with the Empty Vector Control, the Vector Containing *fabK* or *fabX* Expressed from the ATc-Inducible Promoter^a^

	681 MIC (μg/mL)		TLM MIC (μg/mL)	
strain	uninduced	induced	uninduced	induced
empty vector	0.8	0.4	7.8	7.8
FabK overexpression	0.8	>100	7.8	3.9
FabX overexpression	0.8	3.1	7.8	3.9

a Expression was induced with 62.5 ng/mL of ATc (anhydrotetracycline). MICs were determined from three biological replicates.

Third, we generated 681-resistant mutants by serially passaging *F. nucleatum* 23726 in increasing concentrations of compound until there was at least an eightfold increase in the MIC. This corresponded to 4–5 passages across three separate cultures. Mutants isolated from each experiment (i.e., JR1-JR4) had MICs of 3.1–12.5 μg/mL and were genome sequenced to identify key resistance-associated mutations, which were confirmed by Sanger sequencing (Table 3); the parent strain was also sequenced. All genomes were deposited in NCBI under accession number PRJNA1028625. Across the four mutants, three classes of resistance-associated mutations arose as follows: in the ORF for *fabK*, its upstream regulatory region, and/or the upstream region of *C4N14_08920* (which encodes a putative nitronate monooxygenase that is homologous to the *Helicobacter pylori* FabX, a bifunctional dehydrogenase/isomerase, which synthesizes unsaturated fatty acids).^49^ C4N14_08920 aligns with the *H. pylori* FabX, sharing 50.8% identity and 69.6% similarity. C4N14_08920 also possesses the key cysteine residues for the formation of the [4Fe–4S] clusters and residues that are important for FMN binding (Figure S4). Structural modeling of C4N14_08920 further indicated that it was homologous to *H. pylori* FabX (Figure S5). Other mutations acquired during serial passage are reported in Table S3.

**Table 3:** Activity of 681 against Inhibitor-Resistant Mutants of *F. nucleatum* 23726

strain	MIC (μg/mL)^a^	mutations^b^
23726 wild-type	0.4	
JR1	3.1	*fabK* TSS + 1: C > T; FabK: Gly96Ser
JR2	6.3	12 bp deletion upstream of *fabX*
JR3	12.5	*fabK* TSS + 1: C > T
JR4	6.3	12 bp deletion upstream of *fabX*; FabK: Ala132Thr

a MICs were determined from three biological replicates.

b The C > T mutation occurs 1 bp downstream of the *fabK* TSS (transcription start site) determined by Ponath et al.^50^ The 12 bp mutation occurs 99–111 bp upstream of *fabX* start codon, inside a hypothetical protein encoded by *Fn0663*, causing an in-frame deletion of Ser121-Ser124. The full list of mutations occurring during serial evolution in 681 are in Table S3.

### Structural Analysis of Amino Acid Substitutions in *Fn*FabK

Two 681-resistant mutants had mutations in FabK (i.e., Gly96Ser and Ala132Thr in JR1 and JR4, respectively). Homology modeling (Figure 3C) revealed that glycine-96 neighbors the phenylimidazole binding region where the adjacent alanine-97 (backbone amide) forms an H-bond with the imidazole ring of the inhibitor. The Gly96Ser mutation is, therefore, predicted to interfere with the drug-binding pocket. On the other hand, alanine-132 is distantly located from the drug-binding pocket (∼13 Å from the ligand). To better understand the contribution of the Ala132Thr substitution in resistance, we cloned the wild-type alanine-132 and threonine-132 mutants into *F. nucleatum* 23726 under the wild-type *fabK* promoter and determined the 681 MIC (*n* = 4 replicates/test strain). The threonine-132 mutant conferred low-level resistance to 681 (MIC range of 2.5–5 μg/mL) when compared to the wild-type FabK (MIC = 1.3 μg/mL) and the empty vector controls (MIC = 0.6 μg/mL); there was no variation in the replicate MIC values against the wild-type or the empty vector controls. These results suggest that resistance to 681 can develop by direct alterations to FabK, supporting our hypothesis that FabK is the intracellular target.

### Genetic Analysis of Mutations in *fabK* Regulatory Regions

**Figure 4:**
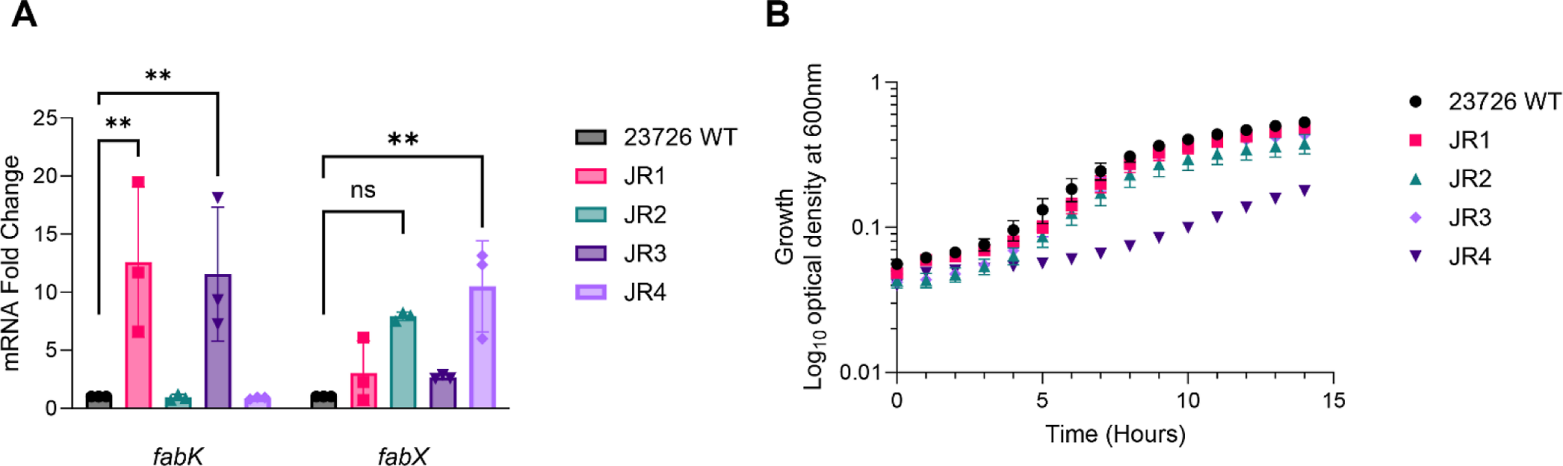
Physiological effects of resistance to 681. Mutants exhibiting resistance to 681 were characterized in terms of expression of *fabK* and *fabX* and effects on growth. (A) RT-qPCR comparing the transcript levels of *fabK* and *fabX* in the wild type and each of the mutants. The data was normalized to 16S rRNA and fold-change is relative to the wild-type strain. The data was obtained from three biological replicates with each having two technical replicates. Statistical analysis was performed using a two-way ANOVA on the dCT values, comparing the mutants to the wild-type strain. ns, *p* > 0.05; **, *p* < 0.01. (B) Growth rates of the 681-resistant mutants, compared to the wild-type strain; data is shown as the mean of 7 biological replicates; error bars indicate the SEM.

In addition to the Gly96Ser mutation, mutant JR1 (MIC = 3.1 μg/mL) evolved a cytosine to thymine mutation upstream of *fabK*. To define the location of this mutation, we compared the mutation site with the genome of *F. nucleatum* 25586 because this strain was previously used to create a comprehensive global transcriptional map of *F. nucleatum* that is found on the Fuso-Base platform (hosted by the Helmholtz Institute for RNA Infection Research).^50^ This identified that the mutation occurred 1 bp from the transcription start site (i.e., TSS + 1) of *fabK*. Further assessment of the mutation region in Fuso-Base and in the bacterial promoter prediction software BPROM revealed that the mutation site was 7 and 30 bp downstream of the −10 (5′ TGATAATAT 3′) and −35 (5′ TTGACA 3′) boxes, respectively. Mutant JR3 (MIC = 12.5 μg/mL) also acquired the TSS + 1 mutation found in JR1. Mutations in regulatory regions of ENR genes are known to enhance transcription, resulting in resistance to inhibitors, as seen with the FabI inhibitor triclosan.^26,51^ Furthermore, as described above, when FabK was overexpressed from the pTET promoter, *F. nucleatum* was resistant to 681. We therefore suspected that the TSS + 1 mutation increased *fabK* mRNA, and this was confirmed by RT-qPCR (Figure 4A). As expected, JR1 and JR3, with the TSS + 1 mutation, showed a significant 7–19-fold increase in *fabK* mRNA transcripts compared to the wild-type transcripts (Figure 4A). In contrast, *fabK* transcript levels for JR2 and JR4, lacking the TSS+1 mutation, resembled those of wild-type (Figure 4A). We confirmed that the increased expression of *fabK* was not due to nonspecific upregulation of FAS-II by showing that there was no effect on *fabG* transcription (*C4N14_09755*; 3-ketoacyl-ACP reductase) (Figure S6).

### Genetic Analysis of Mutations Upstream of *fabX* (*C4N14_08920*)

Mutant JR2 (MIC = 6.3 μg/mL) had a 12 bp deletion (5′ CAAATTATAGTT 3′) that was identified upstream of the putative *fabX* start codon. According to Fuso-Base, *fabX* in *F. nucleatum* 25586 is part of an operon with *Fn0663*(52) (i.e., *C4N14_08915* in strain 23726), a hypothetical protein that is poorly transcribed when compared to *fabX*.^50^ Analysis of the operon in BPROM indicated that there are two transcriptional start sites and two promoters upstream of *fabX*. The first corresponds to the TSS and promoter identified by Ponath et al.^50^ that is upstream of *C4N14_08915*, while the second predicted TSS and promoter are located in *C4N14_08915* at 107 bp upstream of the *fabX* start codon. The 12 bp deletion occurred in *C4N14_08915*, resulting in an in-frame deletion of four amino acids (Ser121-Ser124), which did not affect the second promoter but shortened the distance from the promoter to the start of the gene. This 12 bp mutation also arose in JR4 (MIC = 6.3 μg/mL) that carried the Ala132Thr substitution in FabK. JR2 and JR4 exhibited a 5–13-fold increase in *fabX* transcription when compared to wild-type (Figure 4A); increased expression of *fabX* also did not affect *fabG* transcription (Figure S6). To determine if increased *fabX* expression caused resistance to 681, the gene was expressed from the pTET promoter in wild-type *F. nucleatum* 23726. When *fabX* was induced, the MIC of 681 increased to 3.1 μg/mL (Table 2) when compared to the empty vector (MIC = 0.4 μg/mL). FabX had a significantly lower effect on 681-activity than FabK when both were similarly expressed from the pTET promoter, consistent with FabK being the drug target (i.e., 3.1 μg/mL [for *fabX*] versus >100 μg/mL [for *fabK*]). Interestingly, the pTET overexpression of *fabX* did not impact growth (Figure S7), suggesting that the effects on growth that were seen in JR2 and JR4 (Figure 4B) may be due to the mutation in the hypothetical protein C4N14_08915.

### Antimicrobial Spectrum of Activity of 681

**Figure 5:**
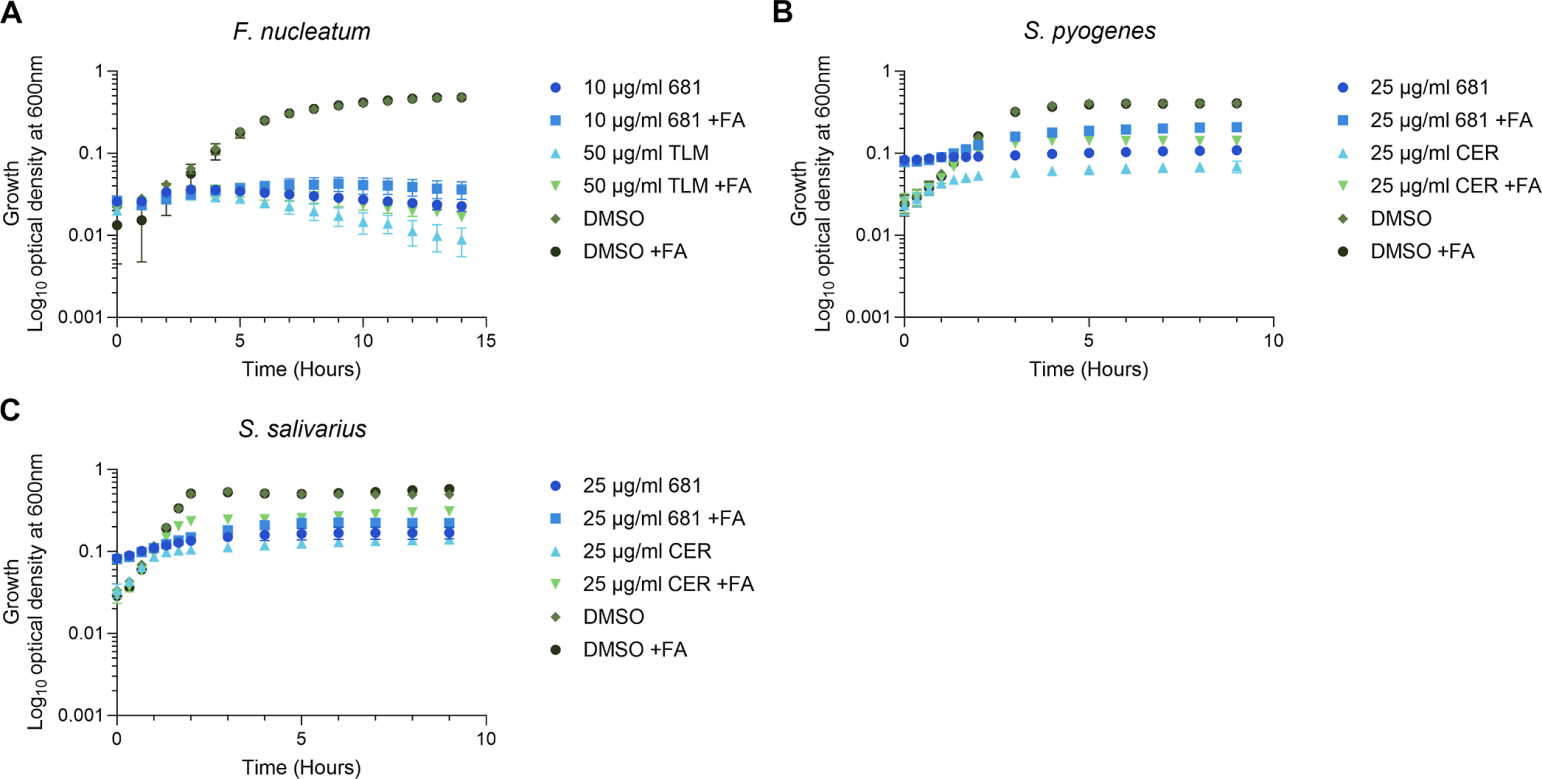
Inability of *F. nucleatum* to bypass FAS-II inhibition when compared to Streptococcal species. The three strains (A) *F. nucleatum* 23726, (B) *S. pyogenes* 19615, and (C) *S. salivarius* HM-121 were grown in FAS-II inhibitors (681, TLM = thiolactomycin, CER = cerulenin) or the DMSO control with or without fatty acids (FAs; myristic acid [C14], palmitic acid [C16], stearic acid [C18], oleic acid [C18:1Δ9], linoleic acid [C18:2Δ9,12], and linolenic acid [C18:3Δ9,12,15]). Concentrations of FAs can be found in Table S2. The data are plotted as the mean of three biological replicates; error bars indicate the SEM.

We evaluated the antimicrobial spectrum of 681 against fusobacteria and other gut flora (Table 4). The compound was inactive (MIC > 100 μg/mL) against representative gut/oral species, including *Bacteroides ovatus* and *Bifidobacterium breve*, but was active (MICs < 1.6 μg/mL) against all tested Streptococci. To examine if *Streptococci* can use environmental fatty acids to bypass 681-inhibition, we tested the commensals *Streptococcus pyogenes* and *Streptococcus salivarius* in comparison to *F. nucleatum* (Figure 5A–C). Figure 5B,C shows that when both species were supplemented with fatty acids, they partly bypassed inhibition by 681. In the presence of 681 and exogenous fatty acids, respectively, *S. pyogenes* and *S. salivarius* showed 4–5-fold and twofold increases in biomass (based on OD_600_nm). In contrast, the same fatty acid composition did not rescue *F. nucleatum* growth, either in 681 or the control thiolactomycin (Figure 5A). This finding supports the above observations that the silencing of *fabK* mRNA was not rescued by fatty acids (Figure 2C). Noteworthily, the protection by fatty acids of *Streptococci* seems weaker when compared to previous work,^26,53^ but this may be due to us using a lower concentration of fatty acids to reflect physiological concentrations in the oral cavity.^45^ Compound 681 was also tested for cytotoxicity against Vero cells (a monkey kidney epithelial cell line) and HCT116 cells (a colonic epithelial cell line). The IC_50_ for 681 was above the maximum tested concentration (>100 μg/mL) in both cell lines, while the cytotoxic controls doxorubicin (Vero) and staurosporine (HCT116) had IC_50_s of 8.1 ± 1.0 μg/mL and 32.6 ± 14.2 μg/mL, respectively. Together, these findings indicate that 681 has promising antibacterial selectivity and minimal cytotoxicity.

**Table 4:** Activity of 681 against a Panel of Species Isolated from the Digestive Tract

species^a^	accession #	ENR	MIC (μg/mL)
*F. nucleatum* 25586	SAMN08706662	FabK	0.8
*F. vincentii* 49256	SAMN02470057	FabK	1.6
*F. periodonticum* 33693	SAMN00008828	FabK	0.8
*F. periodonticum* 2_1_31	SAMN02463707	FabK	0.8
*F. necrophorum* 25286^b^	SAMN10697414	FabK	6.3–12.5
*Streptococcus salivarius*^c^	SAMN00002153	FabK	<1.6
*Streptococcus sanguinis*^c^	SAMN00253296	FabK	<1.6
*Streptococcus mitis*^c^	SAMN02415617	FabK	<1.6
*Streptococcus* sp. CMW7705B^c^	SAMN03956209	FabK	<1.6
*Streptococcus* sp. SPAR10^c^	SAMN00811524	FabK	<1.6
*Streptococcus downei*^c^	SAMN00115114	FabK	<1.6
*Streptococcus pyogenes*^c^	SAMN02905033	FabK	<1.6
*P. gingivalis* F0569^d^	SAMN02436724	FabK	>100
*P. gingivalis* F0185	SAMN02436815	FabK	6.3
*P. gingivalis* F0566	SAMN02436881	FabK	3.1
*P. gingivalis* 33277	SAMD00060922	FabK	>100
*Prevotella denticola*	SAMN00031760	FabI	>100
*Propionibacterium acidifaciens*	SAMN02436184	FabI	6.3
*Clostridium* sp. MSTE9^c^	SAMN00828787	FabK	>100
*Actinomyces* F0386	SAMN00189093	Type I FAS	>100
*Bacteroides ovatus*	SAMN02463791	FabI/FabK	>100
*Bifidobacterium breve*	SAMN00008778	Type I FAS	>100
*E. faecalis* FA 2–2^c^	SAMN22569088	FabI/FabK	>100
*E. faecalis* Δ*fabK*^c^		FabI	>100
*E. faecalis* Δ*fabI*^c^		FabK	<0.1

a Source information can be found in Table S4; the encoded ENR and major FAS-II regulators of each species are indicated. If empty, then no regulator was identified. MICs were determined from three biological replicates.

b The active site of *F. necrophorum* 25286 FabK differs from *F. nucleatum* FabK by the former having an alanine at position 119 instead of a proline in the latter.

c These strains contain the FabT regulator.

d Incomplete inhibition was observed, with a trailing growth effect from 6.3 to 100 μg/mL; hence, the MIC for complete inhibition was >100 μg/mL; FabK of *Pg* strain F0569 is 100% identical to that of strain 33277.

## Conclusions

Narrow-spectrum antimicrobial agents have become increasingly attractive therapeutic concepts for diseases that are characterized by an underlying dysbiosis, infections involving monospecies, and to avoid collateral damage to the gut microbiome.^54,55^ This concept has been focused on ESKAPE pathogens, *C. difficile*, and *Mycobacterium tuberculosis*.^55^ Building on our previous studies establishing *Cd*FabK as a drug target,^26,36^ we identified that *F. nucleatum* FabK is also a target for FabK inhibitors. We presented several lines of evidence that *Fn*FabK is a drug target, including the inability to delete *fabK*; the observed attenuation of growth due to gene silencing of *fabK*; its druggability with inhibitors (e.g., 681 and 701); and the demonstration that 681 targets the enzyme intracellularly. When considered together, the above-mentioned results strongly support our hypothesis that *fabK* is essential to *F. nucleatum* growth under the media conditions tested, with or without exogenous host fatty acids at 37 °C. During these studies, we discovered that *F. nucleatum* encodes a homologue of *H. pylori* FabX and that it can partly compensate for FabK inhibition when dysregulated, as 681-resistant mutants arose by overexpressing *fabX*. FabX is likely involved in the synthesis of unsaturated fatty acids, but since cells require saturated fatty acids to make phospholipids then it is inconceivable that FabX can replace the role of FabK. 681-resistant mutants also arose from amino acid substitutions in FabK and overexpression of *fabK* due to mutations in the promoter region. Supplementation with exogenous fatty acids did not protect *F. nucleatum* against FabK inhibition or the FabB/FabF inhibitor thiolactomycin, meaning that the organism is unlikely to bypass FAS-II inhibition. This is unsurprising given the importance of FAS-II intermediates to the synthesis of Gram-negative outer membranes.^29^ Based on the MICs of 681 against different commensal species, we predict that *Fn*FabK inhibitors could counteract *F. nucleatum* without significantly causing collateral damage. However, further work is needed to examine the efficacy of *Fn*FabK inhibitors in a disease context, such as colorectal cancer or periodontal disease, and their effect on microbiomes. Nonetheless, 681 could represent a good starting point for chemical optimization to discover drug candidates for infections involving *F. nucleatum*.

## Materials and Methods

### Strains and Growth Conditions

*F. nucleatum* subsp. *nucleatum* ATCC 23726 was routinely cultured in Columbia broth or Columbia agar with 5% v/v defibrinated sheep’s blood (HemoStat Laboratories); thiamphenicol (5 μg/mL) was added to maintain plasmids. All strains, plasmids, and media conditions are in Tables S4 and S5. Anaerobic cultures were grown at 37 °C in a Don Whitley A35 anaerobic chamber; aerobic cultures were grown at 37 °C in 5% CO_2_ in a humidified incubator. When required, media was supplemented with fatty acids that were from the following sources [Sigma (oleic acid and stearic acid), Acros Organics (linoleic acid, myristic acid, and palmitic acid), and Chem Impex (linolenic acid)].

### FAS-II Inhibitors

The synthesis of 681 and all other FabK inhibitors was fully described by Norseeda et al.^36^ Compound structures are listed in Table S6. Cerulenin was from the Cayman Chemical Company, and thiolactomycin was from Toronto Research Chemicals.

### Bioinformatics Analysis

This was performed by using the *F. nucleatum* genome of strains 23726 and 25586. KEGG and GenBank annotations were reviewed, with confirmation of annotated genes by searching with both DELTA-BLAST and PHYRE2. *Fn*FabK was further confirmed as the active site residue, His-145, aligned to the active histidine of the *E. faecalis* FA2–2 FabK (accession no. SAMN22569088). *Fn*FabK also had identical residues for each of the FMN binding residues (Gly-19, Ala21, Asn-69, Glu-137, Gly142, Gly-170, Gln189, Gly-191, and Thr-192), except Gly-19, which is an alanine in *Fn*FabK. Since FabK was identified as the sole ENR of *F. nucleatum* 23726, the analysis was expanded to investigate the ENRs of other *Fusobacterium* spp.

### Genetic Manipulation of *F. nucleatum*

The transformation of *F. nucleatum* 23726 was performed via electroporation as previously described,^41^ except that Columbia broth was used. (i) Antisense vector: *F. nucleatum* gene silencing vector, pHFK2, was developed by inserting the ATc inducible paired-termini antisense sequences from the clostridial vector pMSPT into pCWU6, a *F. nucleatum* vector.^56^ pCWU6 was amplified by PCR (CloneAmp polymerase) and recombined with the *Bam*HI and *Kpn*I fragments of pMSPT containing the Tet promoter and paired-termini sequences.^26^ Antisense nucleotides targeting either 50 bp upstream and downstream of the *fabK* start codon (FabKAS1) or 25 bp upstream and 75 bp downstream (FabKAS2) were cloned into SphI and XhoI restriction sites of pHFK2. (ii) Complementation vectors: these were generated by PCR amplifying the *fabK* promoter and gene and introducing *Kpn*I and *Hin*dIII restriction sites at 5′ and 3′ ends, respectively. Purified PCR amplicons were inserted into the *Kpn*I and *Hin*dIII sites of pCWU6. (iii) Overexpression vectors: the corresponding gene, *fabK* or *fabX*, was amplified via PCR, introducing *Hin*dIII or SacI restriction sites to the 5′ end, respectively. *Bam*HI restriction sites were introduced to the 3′ end of both. Purified PCR amplicons were inserted into the respective restriction sites of pHFK2. (iv) Allelic exchange vector: vector, pCWU8-FabK, was generated using a previously described protocol,^41,56^ whereby 1-kb regions upstream and downstream of *fabK* were cloned into the suicide vector pCWU8 using a Gibson Assembly Cloning Kit (New England Biolabs). The recombinant vector was transformed into strain *F. nucleatum* 23726Δ*galK*, and successful integrants were collected that were resistant to thiamphenicol (5 μg/mL). Since the suicide vector expresses GalK, which is toxic in the presence of 2-deoxy-d-galactose (2-DG), the compound 2-DG (0.25% w/v) was added to counter-select for mutants that underwent a second homologous recombination. Ten resulting colonies were then tested for deletion of *fabK* via PCR, using primers in Table S7. The experiment was independently performed twice for a total of 20 colonies screened for *fabK* deletion. All experiments were done anaerobically at 37 °C in tryptic soy broth supplemented with 1% w/v peptone and 0.25% w/v autoclaved l-cysteine hydrochloride (TSPC) or TSPC agar with thiamphenicol or 2-DG.

### Determination of Minimum Inhibitory Concentration (MICs)

Compounds were twofold serially diluted (100–0.2 μg/mL) across a 96-well plate in 100 μL of media used for the specific organism (Table S4). For anaerobes, plates were incubated anaerobically at 37 °C for 1 h and then inoculated with approximately 10^5^ to 10^6^ CFU/well. Plates were incubated at 37 °C for up to 48 h, and MICs were read by visual inspection.

### Growth Kinetics

To plates containing twofold serial dilutions of compounds, exponential phase cultures were added to a final OD_600_nm of 0.1. Plates containing *F. nucleatum* in Columbia broth were incubated at 37 °C inside of a Tecan Infinite 200 Pro, which was inside an anaerobic chamber; growth kinetics for *Streptococcus* spp. in brain heart infusion broth were done aerobically in the same Tecan instrument.

### Transcription Analysis

For antisense analysis, mid-logarithmic cultures (OD_600_nm 0.45–0.65) in Columbia broth (75 mL) were split into two 30 mL aliquots and treated with either 62.5 ng/mL ATc or vehicle (ethanol). Aliquots (5 mL) were recovered at time 0 and at 1 and 3 h after treatment with ATc or vehicle. The aliquots were treated with a recipe of RNALater,^57^ and cell pellets were collected by centrifugation at 4000*g* for 10 min at 4 °C and stored overnight at −80 °C. After mechanical cell lysis, RNA was isolated using the Qiagen RNEasy mini kit. cDNA was synthesized from 200 ng of RNA using qScript cDNA SuperMix (Quanta Biosciences), and RT-qPCR was performed with SsoAdvanced Universal SYBR Green Supermix (Bio-Rad) in an Applied Biosystems QuantStudio 6 Flex. Results were calculated using the 2^–ΔΔCT^ method^58^ and normalized to the 16S rRNA. Gene expressions in mutants were analyzed similarly. Primers used for RT-qPCR analysis can be found in Table S7.

### Generation of 681-Resistant Mutants

Overnight cultures of *F. nucleatum* 23726 were diluted 1:50 in fresh Columbia broth, which was used to inoculate 96-well plates containing twofold serial dilutions of compound 681. After 48 h of incubation, cultures were recovered from wells one dilution below the MIC, and this was used to establish the inocula for the subsequent passage into 96-well plates containing 681. This process was repeated until cultures grew at 8× the original MIC for two consecutive passages. Cultures were then plated onto Columbia blood agar plates and incubated for 72 h at 37 °C. Single colonies were collected into Columbia broth with 0.39 μg/mL of 681 and cultured for up to 48 h and then plated onto selective Columbia blood agar plates to further purify putative 681-resistant colonies.

### Genome Sequencing and Analysis

DNA was extracted from overnight cultures (15 mL) using the Qiagen QIAamp DNA mini kit according to the manufacturer’s protocol. Whole genome sequencing was performed by SeqCenter as follows. Sample libraries were prepared using the Illumina DNA Prep kit and IDT 10 bp UDI indices and sequenced on an Illumina NextSeq 2000, producing 2 × 151 bp reads. Demultiplexing, quality control, and adapter trimming were performed with bcl-convert* (v3.9.3) [*bcl-convert: a proprietary Illumina software for the conversion of bcl files to basecalls]. FastQ files were uploaded to the Bacterial and Viral Bioinformatics Resource Center. First, the parent strain was annotated using the Genome Annotation tool. Next, the variation analysis tool was used to identify genetic differences in the mutant genomes. The identified mutations were confirmed via sanger sequencing.

### Protein Expression and Purification

Codon-optimized *F. nucleatumfab*K was cloned into the pET15b (N-term hexa-HIS tag) at NdeI and *Bam*HI restriction sites by Azenta Life Sciences, and the vector was transformed into *Escherichia coli* XL2-Blue for storage and BL21(DE3) for expression. Cells were grown to an OD_600_ of ∼0.6 in 500 mL of Terrific Broth with 100 μg/mL ampicillin at 37 °C with shaking at 250 rpm; 0.1 mM isopropyl β-d-1-thiogalactopyranoside was used to induce expression, while 0.5 mM flavin mononucleotide (FMN) was also added to improve protein stability and yield. Cells were then incubated for an additional 18 h at 18 °C and 220 rpm before being harvested by centrifugation at 10,000 rpm for 15 min at 4 °C. Pellets were resuspended in lysis buffer containing 50 mM Tris pH 8.0, 100 mM NH_4_Cl, 10% v/v glycerol, 100 μM FMN, 5 mM imidazole, 0.5 mg/mL lysozyme, 0.5% v/v Triton-X 100, 5 mM MgCl_2_, 25 mM sucrose, 2 mM dithiothreitol (DTT), 1 mg/mL DNase, and 1.5 mL protease inhibitor cocktail per 100 mL. For every gram of cells, 10–15 mL of lysis buffer was used and stirred at 4 °C for 1 h before being sonicated at 50% amplitude for a total of 8 min using a cycle of 8 s on and 24 s off. The lysate was centrifuged at 18,000 rpm at 4 °C for 15 min, after which the supernatant was passed through a 0.45 mm filter. The first step of purification uses His Trap affinity chromatography on a 5 mL HP column (Cytiva Life Sciences). The binding buffer contained 50 mM Tris pH 8.0, 100 mM NH_4_Cl, 10% v/v glycerol, 100 μM FMN, 5 mM imidazole, and 2 mM DTT, and the elution buffer contained the same components with an increased imidazole concentration of 250 mM. Eluted protein was further purified by gel filtration on Superdex 200 pg (Cytiva Life Sciences) using a running buffer containing 50 mM HEPES pH 8.0, 300 mM NH_4_Cl, 10% v/v glycerol, 2 mM DTT, and 100 μM FMN. After purification, concentrated protein was stored at −80 °C in 35% v/v glycerol.

### *Fn*FabK Enzymatic Assay

The *Fn*FabK assay was conducted via the following protocol: reactions were carried out at 25 °C in assay buffer (100 mM HEPES pH 8.0, 500 mM NH_4_Cl, 10% v/v glycerol) with 150 μM crotonyl-CoA (butenoyl-CoA) and 160 μM NADH. FabK enzyme was diluted using 2.5 mg/mL gamma globulin in assay buffer to a working stock of 30 nM and was incubated with 681 threefold serial dilutions ranging from 100 μM to 1.69 nM. Incubation lasted for a total of 10 min before the addition of crotonyl-CoA substrate, and the reaction was initiated by the addition of NADH for a final assay volume of 100 μL. The oxidation of NADH to NAD+ was measured by tracking fluorescence (340 nm/460 nm) with a Biotek Synergy H1 microplate reader in 15 s intervals for a total of 10 min to monitor the rate of reaction. The reaction was conducted in Greiner Bio-One 384-well μClear Bottom Polystyrene Microplates. For IC_50_ calculations, linear slopes were calculated from the first 5 min and used to determine the reaction rates. Measurements were conducted in triplicate, and IC_50_’s were calculated via GraphPad Prism 9.1.2 using four-parameter logistic (Hill) curve analysis using equation *Y* = bottom + (top – bottom)/[1 + 10 (Log IC_50_ – *X*) × Hill Slope], where *X* is the logarithm of the dose and *Y* is the response. The assay concentrations of the cofactor, NADH, and substrate, crotonyl-CoA, were also determined via GraphPad Prism 9.1.2 using the substrate inhibition model to determine *K*_m(app)_.

### FnFabK 681 Homology Modeling

The homodimer homology model of *Fn*FabK was generated from the *S. pneumoniae* FabK experimental structure (PDB ID: 2Z6J),^47^ using Prime software^59,60^ in the 2023 Schrödinger modeling suite (Schrödinger Release 2023-1: Prime, Schrödinger, LLC, New York, NY, 2023), and included placement of the FMN cofactor and inhibitor compound. The faster, knowledge-based model-building method was selected due to the high sequence similarity. Default values were used for other settings. Structure and loop position refinements were performed by using iterative energy calculations.

### Cytotoxicity

This was done as described^36^ using colorectal carcinoma HCT116 and Vero epithelial cells cultured at 37 °C in 5% CO_2_ in Dulbecco’s modified Eagle’s medium (DMEM) with 4.5 g/L glucose and l-glutamine. The medium was supplemented with 10% v/v FetalGro (Rocky Mountain Biologicals). Approximately 25,000/well were aliquoted into black 96-well plates (Greiner Bio-One) and incubated overnight, before being resuspended in DMEM supplemented with 20% Gibco KnockOut Serum Replacement (Thermo Fisher Scientific). Compounds (100–1.6 μg/mL) were then added to the cells and incubated for 24 h at 37 °C and 5% CO_2_. Cell viability was analyzed using resazurin as described.^36^

### Statistical Analysis

All statistical analysis was performed using GraphPad Prism version 10.1.1.

## Supporting Information Available

Gene nomenclature for *F. nucleatum* ATCC 25586 and the corresponding gene name in *F. nucleatum* ATCC 23726, composition of fatty acid mixture supplemented during bypass experiments, other mutations identified in the 681-resistant *F. nucleatum* 23726 mutants, strains and corresponding media used in this study; plasmids used in this study, structures of phenylimidazole FabK inhibitors, primers used in this study, alignment of the *F. nucleatum* 23726 FabK to the *C. difficile* FabK, schematic overview of cloning strategy for knockdown vector, growth rates of *F. nucleatum* antisense strains when induced with 62.5 ng/mL ATc and supplemented with 0.1% Tween-80, alignment of C4N14_08920 to *H. pylori* FabX, structural homology of C4N14_08920 to *H. pylori* FabX, *fabG* transcript levels of 681-resistant mutants, and growth rates of *F. nucleatum* FabK or FabX overexpression strains (PDF)
