# Supplementary material for: Evaluation of Fusobacterium nucleatum Enoyl-ACP Reductase (FabK) as a Narrow-Spectrum Drug Target
Source: ACS Infect Dis. 2024 Apr 10;10(5):1612–23. doi: 10.1021/acsinfecdis.3c00710 (PMC11091888; doi:10.1021/acsinfecdis.3c00710)
Supplement: Supplementary file 1 — id3c00710_si_001.pdf [file id3c00710_si_001.pdf]

Supporting Information

Evaluation of *Fusobacterium nucleatum* Enoyl-ACP Reductase (FabK) As A Narrow-Spectrum Drug Target

Jacob T. Rutherford;[1] Kristiana Avad;[2] Chetna Dureja;[1] Krissada Norseeda;[3] Bibek GC;[4] Chenggang Wu;[4] Dianqing Sun;[3] Kirk E Hevener;[2] Julian G. Hurdle[1]

[1]Center for Inflammatory and Infectious Diseases, Texas A&M Health Science Center Institute of Biosciences and Technology, Houston, TX, 77030, USA.

[2]Department of Pharmaceutical Sciences, College of Pharmacy, University of Tennessee Health Science Center, Memphis, TN, 38163, USA.

[3]Department of Pharmaceutical Sciences, The Daniel K. Inouye College of Pharmacy, University of Hawaii at Hilo, Hilo, HI, 96720, USA.

[4]Department of Microbiology & Molecular Genetics, McGovern Medical School, University of Texas Health Science Center at Houston, Houston, TX, 77030, USA.

Corresponding author: Email: jhurdle@tamu.edu

Table of Contents

Content	Description	Page
Table S1	Gene nomenclature for *F. nucleatum* ATCC 25586 and the corresponding gene name in *F. nucleatum* ATCC 23726	S3-S4
Table S2	Composition of fatty acid mixture supplemented during bypass experiments	S4
Table S3	Other mutations identified in the 681 resistant *F. nucleatum* 23726 mutants	S5
Table S4	Strains and corresponding media used in this study	S6-S7
Table S5	Plasmids used in this study	S7
Table S6	Structures of phenylimidazole FabK inhibitors	S8-S14
Table S7	Primers used in this study	S15-S17
Figure S1	Alignment of the *F. nucleatum* 23726 FabK to the *C. difficile* FabK	S18
Figure S2	Schematic overview of cloning strategy for knockdown vector	S19
Figure S3	Growth rates of *F. nucleatum* anti-sense strains when induced with 62.5 ng/ml ATc and supplemented with 0.1% Tween-80	S20
Figure S4	Alignment of C4N14_08920 to *H. pylori* FabX	S21
Figure S5	Structural homology of C4N14_08920 to *H. pylori* FabX	S22
Figure S6	*fabG* transcript levels of 681 resistant mutants	S23
Figure S7	Growth rates of *F. nucleatum* FabK or FabX overexpression strains	S24
SI References		S25

TABLES

Table S1. Gene nomenclature for *F. nucleatum* ATCC 25586 and the corresponding gene name in *F. nucleatum* ATCC 23726

***F. nucleatum* ATCC 25586**	***F. nucleatum* ATCC 23726**	**Gene Function**
Fn0174	C4N14_04250	Enoyl-[acyl-carrier-protein] reductase II (FabK)
Fn0150	C4N14_04130	Acyl-carrier protein
Fn0664	C4N14_08920	Nitronate monooxygenase (putative FabX)
Fn0663	C4N14_08915	Hypothetical protein
Fn0624	C4N14_09120	Methionine transporter (MetT)
Fn0625	C4N14_09115	Putative aminotransferase
Fn1272	C4N14_05770	AcrR family transcriptional regulator
Fn0408	C4N14_10115	Acetyl-coenzyme A carboxylase carboxyl transferase subunit beta
Fn0409	C4N14_10110	Acetyl-coenzyme A carboxylase carboxyl transferase subunit alpha
Fn0149	C4N14_04125	Malonyl-CoA-[acyl-carrier-protein] transacylase (FabD)
Fn0151	C4N14_04135	3-oxoacyl-[acyl-carrier-protein] synthase II (FabF)
Fn0148	C4N14_04120	3-oxoacyl-[acyl-carrier-protein] synthase III
Fn1850	C4N14_03815	3-oxoacyl-[acyl-carrier-protein] synthase III
Fn0216	C4N14_04475	3-oxoacyl-[acyl-carrier protein] reductase
Fn0494	C4N14_09755	3-oxoacyl-[acyl-carrier protein] reductase (FabG)

Fn0899	C4N14_07675	3-oxoacyl-[acyl-carrier protein] reductase
Fn0594	C4N14_09260	3-hydroxyacyl-[acyl-carrier-protein] dehydratase (FabZ)

Table S2. Composition of fatty acid mixture used to supplement growth under anti-sense inhibition of *fabK* or in compound 681.

Fatty acid	Abbreviation	Concentration
Myristic	C14:0	100 nM
Palmitic	C16:0	1.5 μM
Stearic	C18:0	1 μM
Oleic	C18:1Δ9	3 μM
Linoleic	C18:2Δ9,12	1 μM
Linolenic	C18:3Δ9,12,15	50 nM

Table S3. Other mutations identified in the 681-resistant *F. nucleatum* 23726 mutants.

Strain	MIC (µg/ml)	FabK TSS[a]	Enoyl-ACP Reductase FabK (*C4N14_04250*)	Acyl-Carrier Protein (*C4N14_04130*)	Transcriptional Regulator AcrR Family (*C4N14_05770*)	Hypothetical Protein (*C4N14_08915*)	Methionine Transporter MetT (*C4N14_09120*)	Putative Aminotransferase (*C4N14_09115*)
**Experimental Replicate 1**								
**JR 1**	12.5	C>T	Gly96Ser		Ile202fs		Gly393Ser	
**Experimental Replicate 2**								
**JR 2**	3.1					Ser121-Ser124del		Gly98_Val99fs
**JR 4**	6.3		Ala132Thr			Ser121-Ser124del		Gly98_Val99fs
**Experimental Replicate 3**								
**JR 3**	3.1	C>T		Asp54Asn				Gly98_Val99fs

[a]Mutation occurs 1 bp downstream of the *fabK* (enoyl-acyl carrier protein (ACP) reductase II) Transcription Start Site (TSS)

Table S4. Strains and media used in this study

Strain	Species	Media for cultivation	Reference/Source
HM-121	*Streptococcus salivarius*	Brain Heart Infusion (BHI)	BEI Resources
HM-275	*Streptococcus sanguinis*	BHI	BEI Resources
HM-262	*Streptococcus mitis*	BHI	BEI Resources
HM-1296	*Streptococcus sp.* CMW7705B	BHI	BEI Resources
NR-34818	*Streptococcus sp.* SPAR10	BHI	BEI Resources
HM-475	*Streptococcus downei*	BHI	BEI Resources
ATCC19615	*Streptococcus pyogenes*	BHI	ATCC
HM-1072	*Porphyromonas gingivalis* F0569	Brucella Blood Agar with Hemin (5μg/ml) and Vitamin K (10μg/ml) (BHK) and ATCC Medium 2722	BEI Resources
HM-1140	*Porphyromonas gingivalis* F0185	BHK and ATCC Medium 2722	BEI Resources
HM-1141	*Porphyromonas gingivalis* F0566	BHK and ATCC Medium 2722	BEI Resources
ATCC 33277	*Porphyromonas gingivalis* 33277	BHK and ATCC Medium 2722	ATCC
HM-208	*Prevotella denticola*	BHK and ATCC Medium 2722	BEI Resources
HM-8	*Propioinibacterium acidifaciens*	BHK and ATCC Medium 2722	BEI Resources
HM-764	*Clostridium sp.* MSTE9	BHK Agar and BHI Broth	BEI Resources
HM-468	*Actinomyces* F0386	BHK Agar and BHI Broth	BEI Resources
HM-222	*Bacteroides ovatus 3_8_47FAA*	BHK Agar and BHI Broth	BEI Resources
ATCC 15700	*Bifidobacterium breve* 15700	BHK Agar and BHI Broth	ATCC
ATCC 23726	*Fusobacterium nucleatum subsp. nucleatum* 23726	Columbia Blood Agar and Columbia Broth	ATCC
23726 Δ*galK*	*F. nucleatum subsp. nucleatum* 23726 ΔGalK	Columbia Blood Agar and Columbia Broth	[1]
ATCC 25586	*F. nucleatum subsp. nucleatum* 25586	Columbia Blood Agar and Columbia Broth	ATCC

ATCC 49256	*F. nucleatum subsp. vincentii* 49256	Columbia Blood Agar and Columbia Broth	ATCC
ATCC 33693	*F. periodonticum* 33693	Columbia Blood Agar and Columbia Broth	ATCC
HM-42	*F. periodonticum* 2_1_31	Columbia Blood Agar and Columbia Broth	BEI Resources
FA2-2	*Enterococcus faecalis*	BHI with 10g/L Tryptone and 5g/L Yeast Extract (BHI-TY)	[2]
FA2-2 Δ*fabK*	*Enterococcus faecalis* Δ*fabK*	BHI-TY	[2]
FA2-2 Δ*fabI*	*Enterococcus faecalis* Δ*fabI*	BHI-TY	[2]
JR1	*F. nucleatum subsp. nucleatum* 23726_JR1	Columbia Blood Agar and Columbia Broth	This Study
JR2	*F. nucleatum subsp. nucleatum* 23726_JR2	Columbia Blood Agar and Columbia Broth	This Study
JR3	*F. nucleatum subsp. nucleatum* 23726_JR3	Columbia Blood Agar and Columbia Broth	This Study
JR4	*F. nucleatum subsp. nucleatum* 23726_JR4	Columbia Blood Agar and Columbia Broth	This Study

Table S5. Plasmids used in this study.

Plasmid	Description	Reference
pCWU8	Parent vector for the deletion vector	[1, 3]
pCWU8-FabK	FabK deletion vector	This Study
pCWU6	Fuso cloning vector	[1]
pMSPT	Contains pTET for ATc induction	[4]
pHFK2	Empty vector for ATc induction	This Study
pHFK2-FabKAS1	Knockdown vector targeting 50 bp up and down stream of the FabK start codon	This Study
pHFK2-FabKAS2	Knockdown vector targeting 25bp up and 75bp downstream of the FabK start codon	This Study
pCWU6-WTFabK	Wild-type *fabK* and the promoter cloned into pCWU6 at KpnI and HindIII sites	This Study
pCWU6-JR4FabK	The *fabK* gene and promoter from JR4 (A132T mutation) cloned into the KpnI and HindIII sites	This Study
pHFK2-FabK	Overexpression vector for *F. nucleatum* 23726 FabK	This Study
pHFK2-FabX	Overexpression vector for *F. nucleatum* 23726 FabX	This Study

Table S6. Structures of phenylimidazole FabK inhibitors.

Structure	Compound[a]	LogP	LogD (pH 7.4)
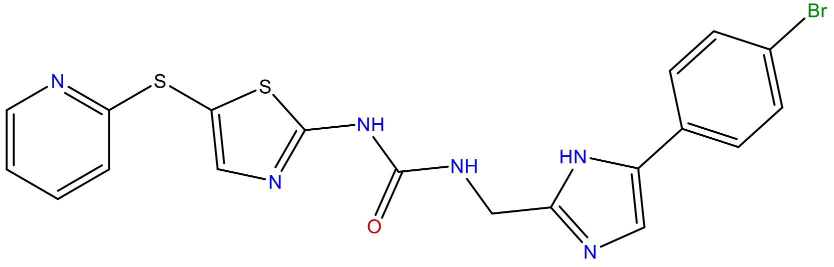	296 (5a)	4.657	4.52
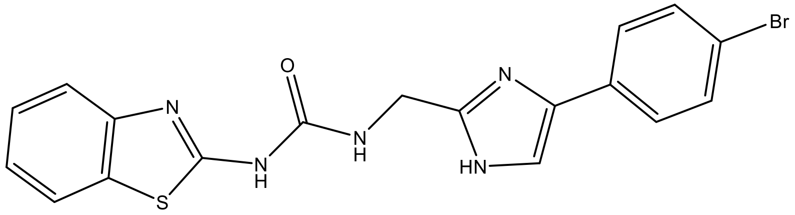	701 (6a)	4.407	4.26
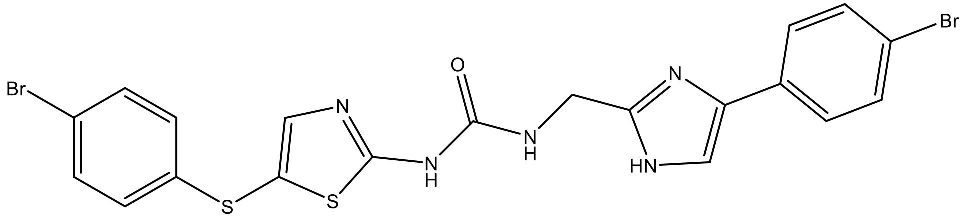	702 (5d)	6.049	5.91

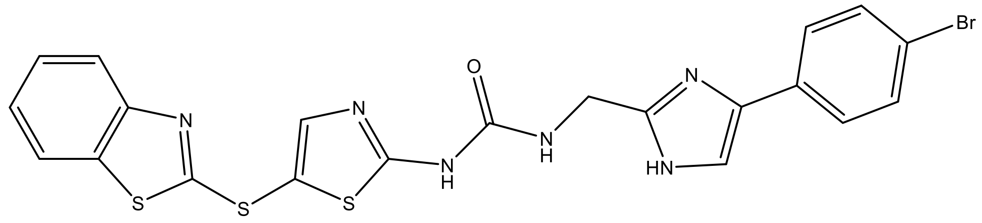	703 (5i)	6.104	5.96
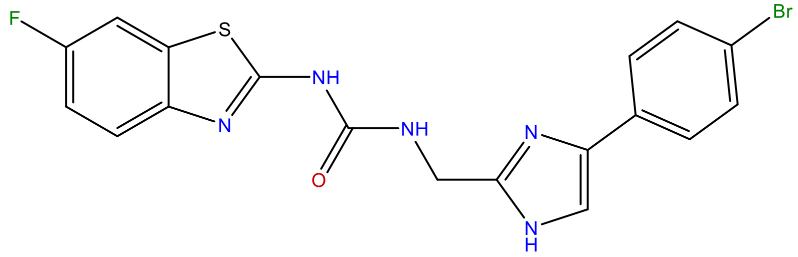	670 (6i)	4.549	4.41
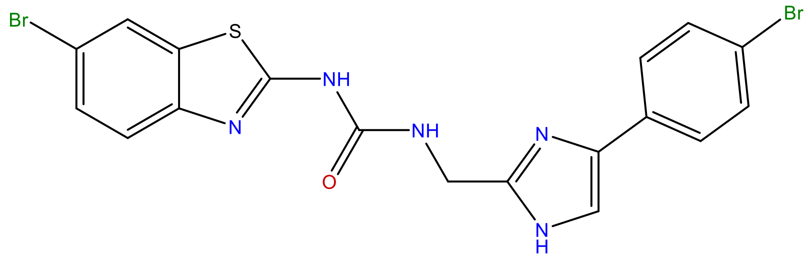	671 (6g)	5.175	5.03

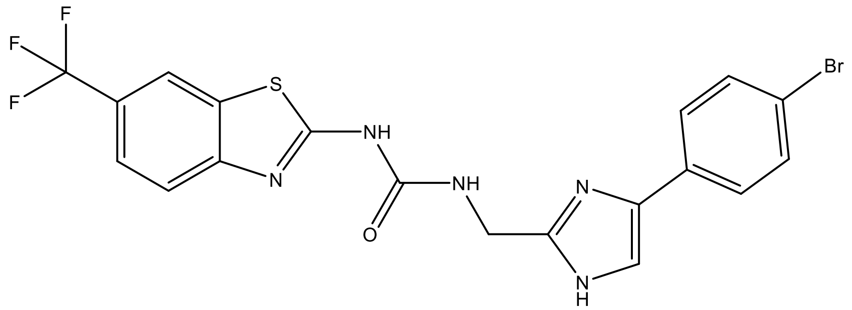	673 (6d)	5.284	5.14
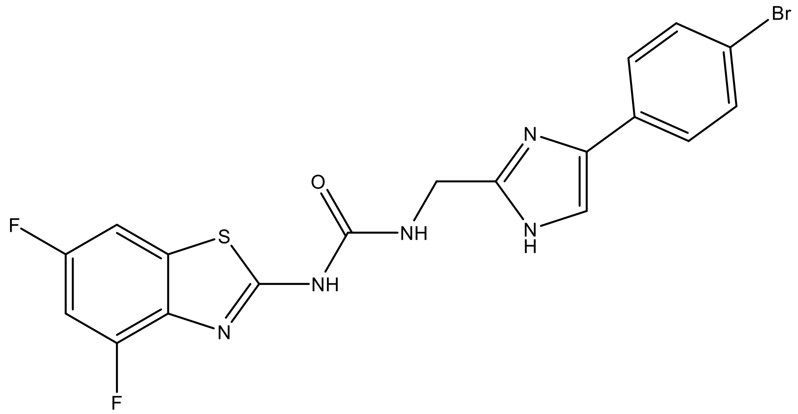	674 (6j)	4.692	4.56
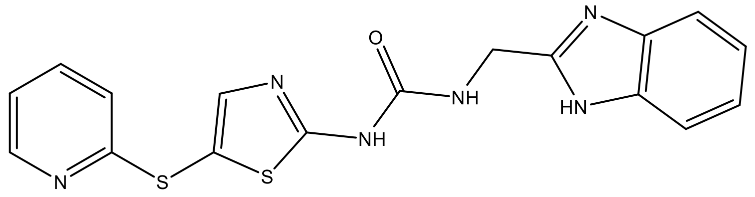	675 (8b)	3.26	3.12

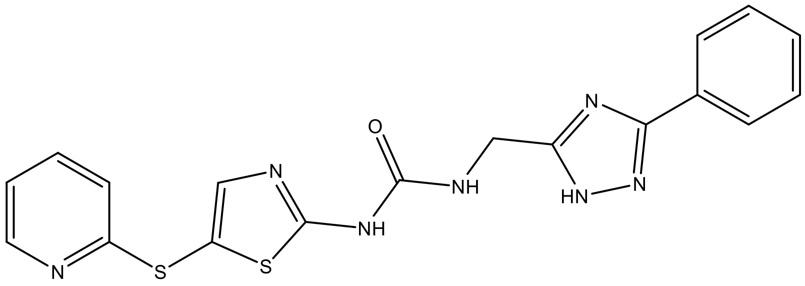	676 (8a)	2.919	2.78
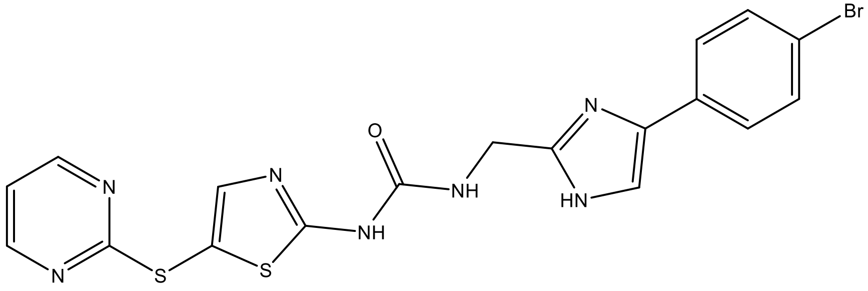	679 (5b)	4.035	3.89
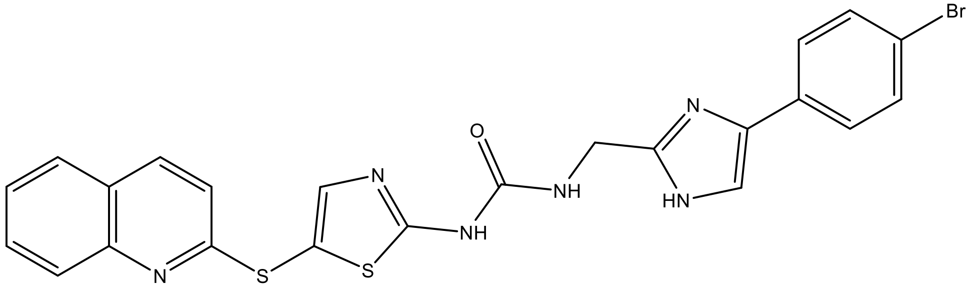	680 (5h)	6.032	5.89

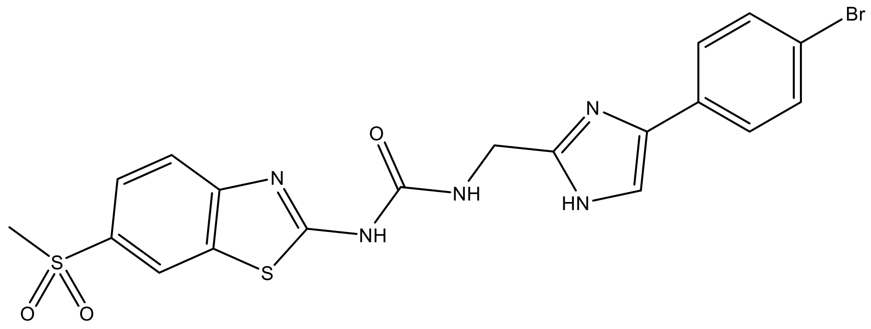	681 (6f)	3.247	3.1
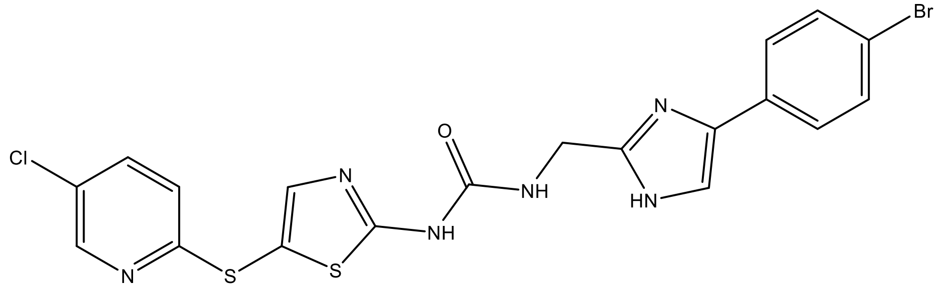	682 (5e)	5.261	5.12

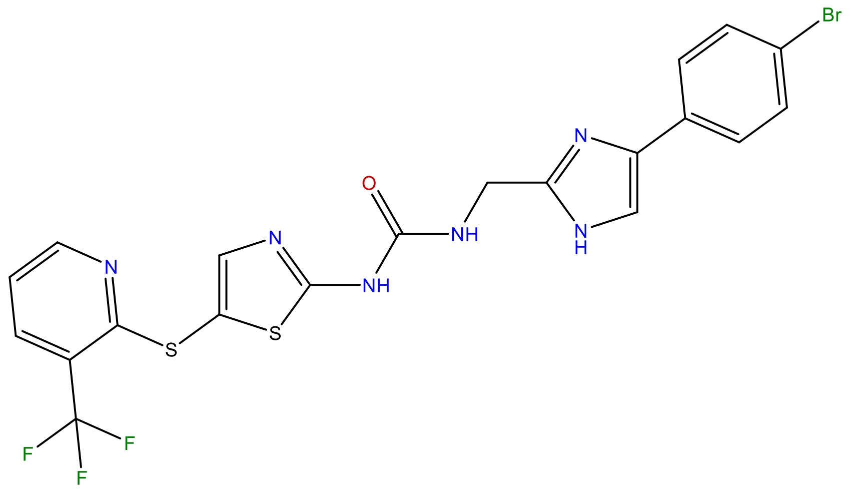	683 (5g)	5.535	5.4
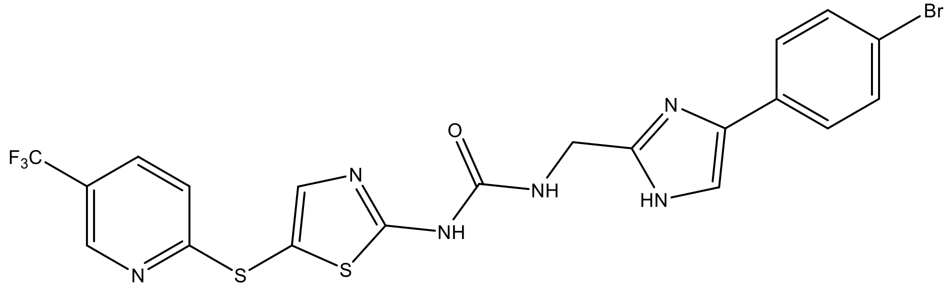	684 (5f)	5.535	5.4

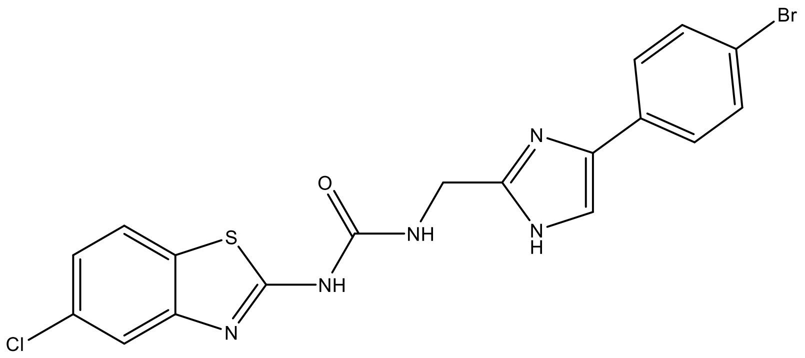	689 (6h)	5.011	4.87
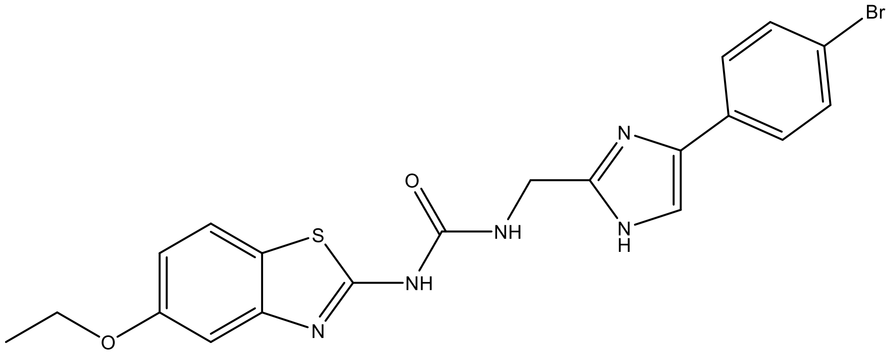	690 (6k)	4.606	4.46

[a]Shown in parentheses is the nomenclature used by Norseeda *et al*.[5] which describes the synthesis of the compounds.

Table S7. Primers used in this study.

Primer	Description	Sequence 5' to 3'
**JRP1**	Linearize pCWU6 for creation of pHFK2	GATAGGTACCACACGACG GCAGGAGTGAGTAGC
**JRP2**		GATAGGATCCGTCGTTCGG CTGCGGCG
**JRP3**	RT-qPCR primers for detecting *fabK* knockdown	TTTTAATTATTAAGATATATG GAGGAGTTTTAATG
**JRP4**		AGGCTCCATACCACCACCA GC
**JRP5**	RT-qPCR primers downstream of knockdown target	GCTAAAGATAGATCAACTA CTGTTACAGGAAC
**JRP6**		GCAACTTGTCCTGACATGA AACTTCC
**JRP7**	Cloning *fabK* and promoter for complementation experiments	GATAGGTACCGCTATGATA CAGATACAAGAATACTTAC AATTAAGAC
**JRP8**		GATAAAGCTTCTTCCACTA AATTCAATAGCCATACG
**JRP9**	Amplification of *fabX* from gDNA for Sanger sequencing	CTATGATGATAGAACTTCAA GACTTATTGC
**JRP10**		GTATCCACAACATATCTTAT AGCCAATGC
**JRP11**	*fabX* RT-qPCR primers	GGTGGACTAGGAACAATAA GTGG
**JRP12**		CTTGAATTTAATGCGTCTG C
**JRP13**	*fabX* Sanger sequencing to confirm mutation	AAAGTTGGAAAATTCTATG AAGAC
**JRP14**		ACTCCTACACCCATTCCAC C
**JRP15**		GCTAATTGATCCCAACTAA CTC

**JRP16**	*fabG* RT-qPCR	ATGAGAATGACAGAAGATCAATGG
**JRP17**		CCATGTAATCCAACAACTGATG
**JRP18**	Linearize parent deletion vector	TAGCCCGCTCAGAAGAACTCGTCAAGAAG
**JRP19**		CATCGATGCATGCGTCGACGTCCATATGCCATGGCCTAGG
**JRP20**	Upstream *fabK* region for deletion	ATGGACGTCGACGCATGCATCGATG ACTTAGTCAATAAAGATAAT ATAAGCG
**JRP21**		TATAACTGCAAGTCTTAAACTCTTTGCTTTTCTAATATTTTCTCTTAAAAG
**JRP22**	Downstream *fabK* region for deletion	TATTAGAAAAGCAAAGAGTTTAAGACTTGCAGTTATAGATGGAGATG
**JRP23**		TTGACGAGTTCTTCTGAGCGGGCTATAACAACCACTGCTTCATCTGCTG
**JRP24**	Confirmation of *fabK* deletion	GAATAAAAAG TGATTTTCTT AAAAGAATAG
**JRP25**		TTAGGATCTAAAGCAATTACTAGTCTATCAT
**JRP26**	Amplification of gene *C4N14_05770* to confirm mutation	GAGATATTGAAAGTGACATGAACTCTGATATAG
**JRP27**		TCCTTCTGAATAGGATAAAGCCTC
**JRP28**	Sanger sequencing of *C4N14_05770*	CCCTTCAAAAGATAAGATGTTAAGTG
**JRP29**		TCCTTCTGAATAGGATAAAGCCTC
**JRP30**	Amplification of gene *C4N14_04130* to confirm mutation	GGGAGGTAAAAATGGGAAAAGTTGC
**JRP31**		GCTCCTAGTCCATGTCCAGTTGCTC

**JRP32**	Sanger sequencing of *C4N14_04130*	GTTGAACAATTAGGAGTGGATGCTG
**JRP33**		CCTGTTACAACAACTCTTTTCATTATTCAC
**JRP34**	Amplification of genes *C4N14_09120* and *C4N14_09115* to confirm mutation	GCTGGTTTAGAAGTTTATACACCTAGTGAG
**JRP35**		CTGTTGGTCTTGGGAAATTATTAGGC
**JRP36**	Sanger sequencing of *C4N14_09120*	CTATGTGGGTCGCTGATATGG
**JRP37**		CTACCAGCAGGATTATGAGGAG
**JRP38**	Sanger sequencing of *C4N14_09115*	GCTTAATTTCAGGGCTTCTATCAG
**JRP39**		TTTCAATGAGTGCCTGCCCAAC
**JRP40**	Cloning of *fabK* for inducible overexpression	GACAAAGCTTAATTATTAAGATATATGGAGGAGTTTTAATG
**JRP41**		GACAGGATCCTTAAATATCCCAATTTTCTAGTCTTC
**JRP42**	Cloning of *fabX* for inducible overexpression	GACAGAGCTCGTAGAATTTAAGGAGGAAAAGATGAAAG
**JRP43**		GACAGGATCCCCTTTTAATATATGGATATTTATTGACAGTC

FIGURES

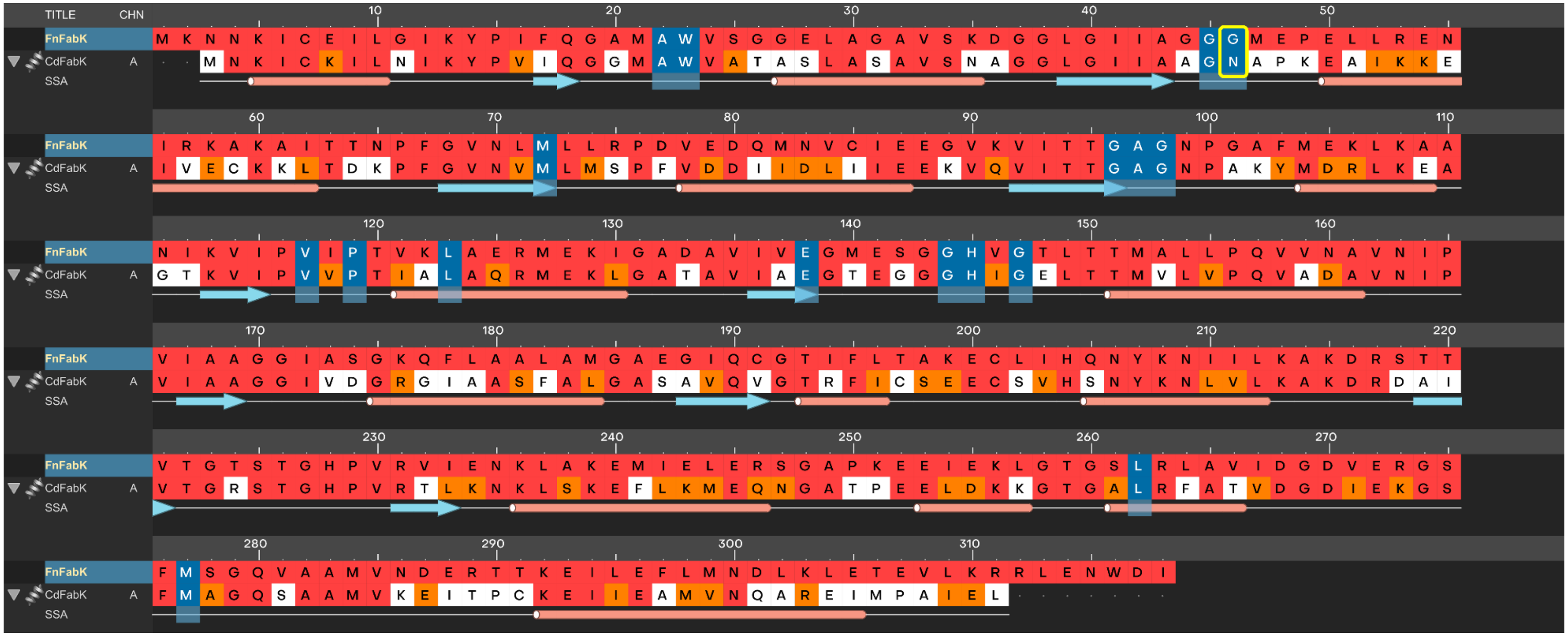

Figure S1. Alignment of the *F. nucleatum* 23726 FabK (*Fn*FabK; AVQ22874.1) to the *C. difficile* FabK (*Cd*FabK; WP_003419125.1). *Fn*FabK and *Cd*FabK share 56% identity and 74% similarity. Residues are highlighted by identity (red) and similarity (orange) to *Fn*FabK. Residues located within 4Å of the active site are highlighted in blue, with the yellow box at residue 46 indicating the sole difference in the active site. Secondary structure is indicated below the alignment; red cylinders are alpha helices and blue arrows are beta strands.

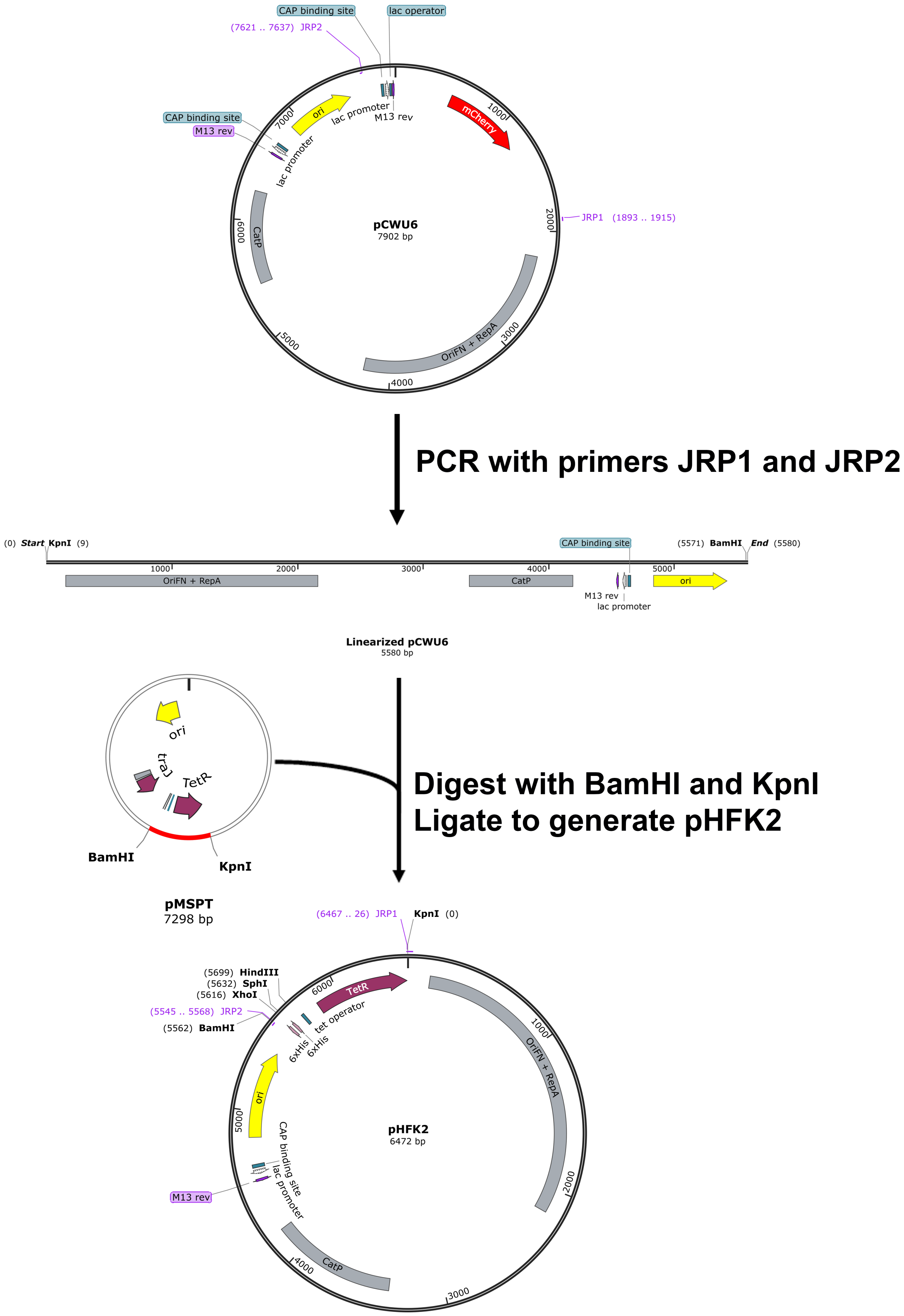
CAP binding site
lac operator
(7621 .. 7637) JRP2
CAP binding site
M13 rev
ori
lac promoter
M13 rev
mCherry
lac promoter
JRP1 (1893 .. 1915)
pCWU6
7902 bp
CatP
OriFN + RepA
PCR with primers JRP1 and JRP2
(0) Start KpnI (9)
CAP binding site
(5571) BamHI End (5580)
OriFN + RepA
CatP
ori
M13 rev
lac promoter
Linearized pCWU6
5580 bp
ori
traJ
TetR
BamHI
KpnI
pMSPT
7298 bp
Digest with BamHI and KpnI
Ligate to generate pHFK2
(6467 .. 26) JRP1
KpnI (0)
(5699) HindIII
(5632) SphI
(5616) XhoI
(5545 .. 5568) JRP2
(5562) BamHI
TetR
tet operator
6xHis
6xHis
OriFN + RepA
ori
pHFK2
6472 bp
CAP binding site
lac promoter
M13 rev
CatP

Figure S2. Schematic overview of the knockdown cloning strategy. The fusobacterial cloning vector pCWU6 was linearized via PCR amplification. Primers were designed to introduce BamHI and KpnI restriction sites at the 5' and 3' end of the vector, respectively. Next, BamHI and KpnI sites were used to transfer the anhydrotetracycline inducible promoter from pMSPT into the PCR product, creating the plasmid pHFK2. Plasmid maps generated using SnapGene® software.

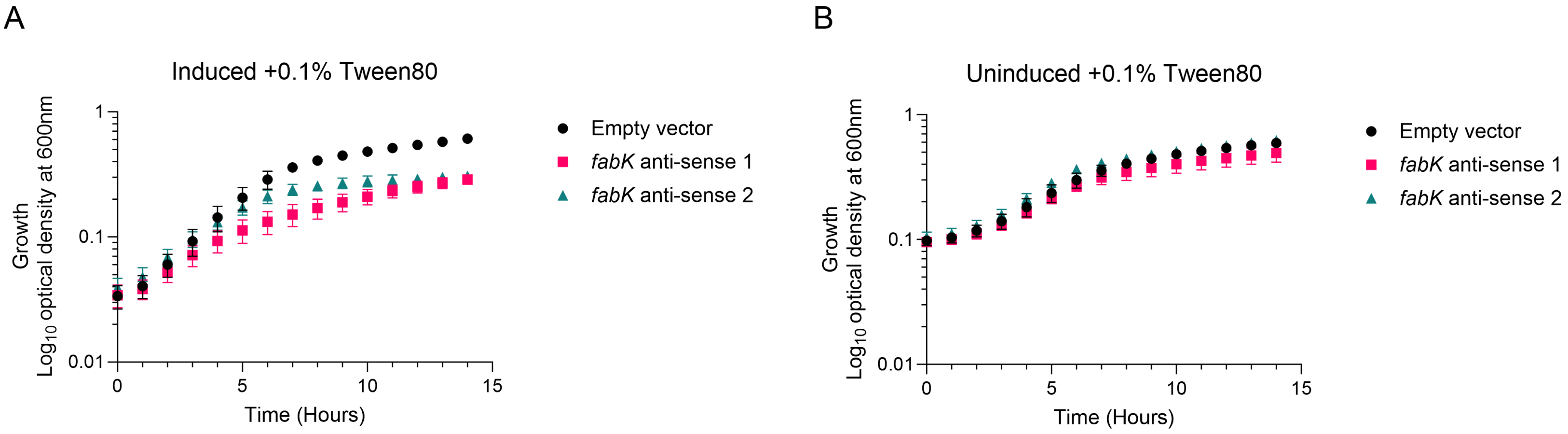

Figure S3. Growth rates of *F. nucleatum* 23726 when expressing anti-senses to *fabK* mRNA or empty vector control (pHFK). The growth media, Columbia broth was supplemented with 0.1% Tween-80 and cells were either treated with 62.5 ng/ml anhydrotetracycline to induce anti-sense expression **(A)** or ethanol (vehicle control) **(B)**. Shown is the mean and SEM of 3 biological replicates. Sequences of the anti-senses are shown as follows:

fabK anti-sense 1

5'AATATTGGATACTTGATTCCTAATATCTCACAAATTTTATTATTTTTCATTAAAACTCCTCCATATATCTTAATAATTAAAATTATTTTTCC TTAATTAT3'

fabK anti-sense 2

5'AGAAACCCAAGCCATAGCTCCTTGAAATATTGGATACTTGATTCCTAATATCTCACAAATTTTATTATTTTTCATTAAAACTCCTCCAT ATATCTTAATA3'

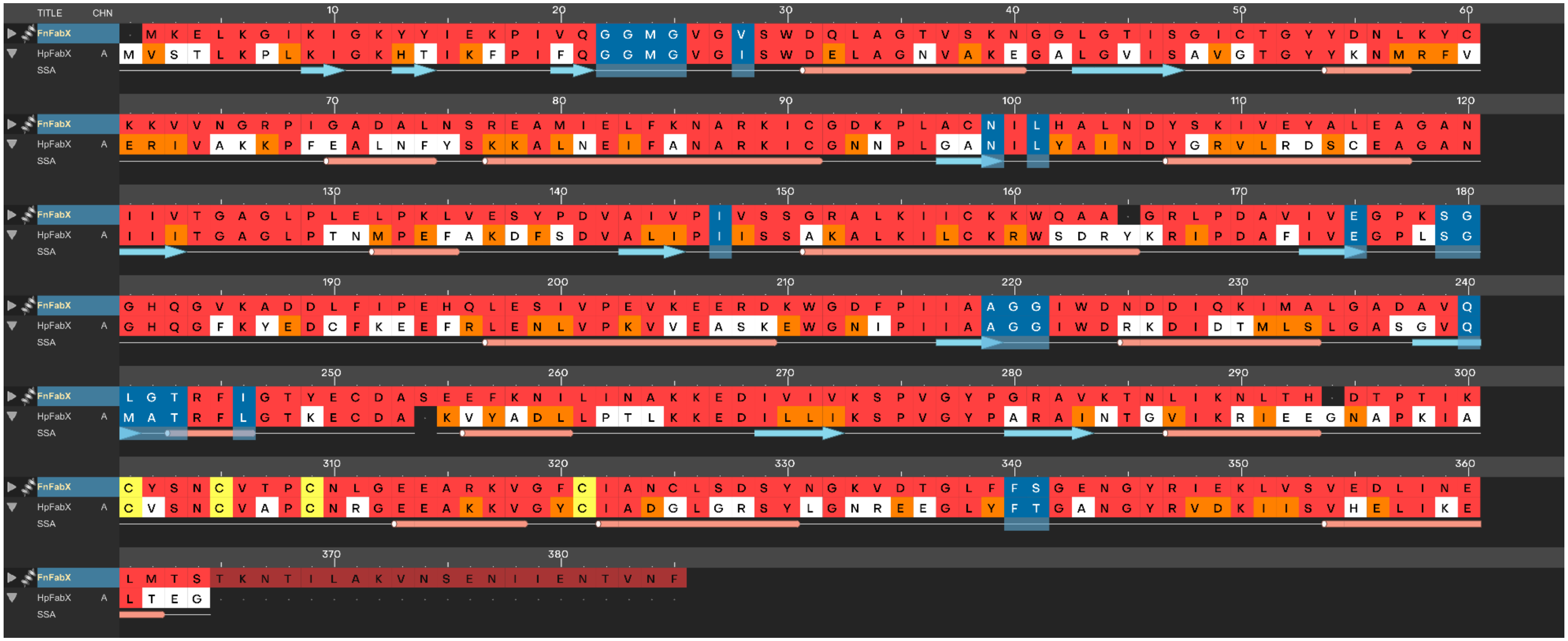

Figure S4. *F. nucleatum* C4N14_08920 is a homologue of *H. pylori* FabX. Alignment of C4N14_08920 to *H. pylori* FabX. C4N14_08920 possesses 50.8% identity and 69.6% similarity to the *H. pylori* FabX. Shown in red are identical residues, orange indicates similar residues. The residues involved in FMN binding are highlighted in blue, and the four cysteine residues are shown in yellow.

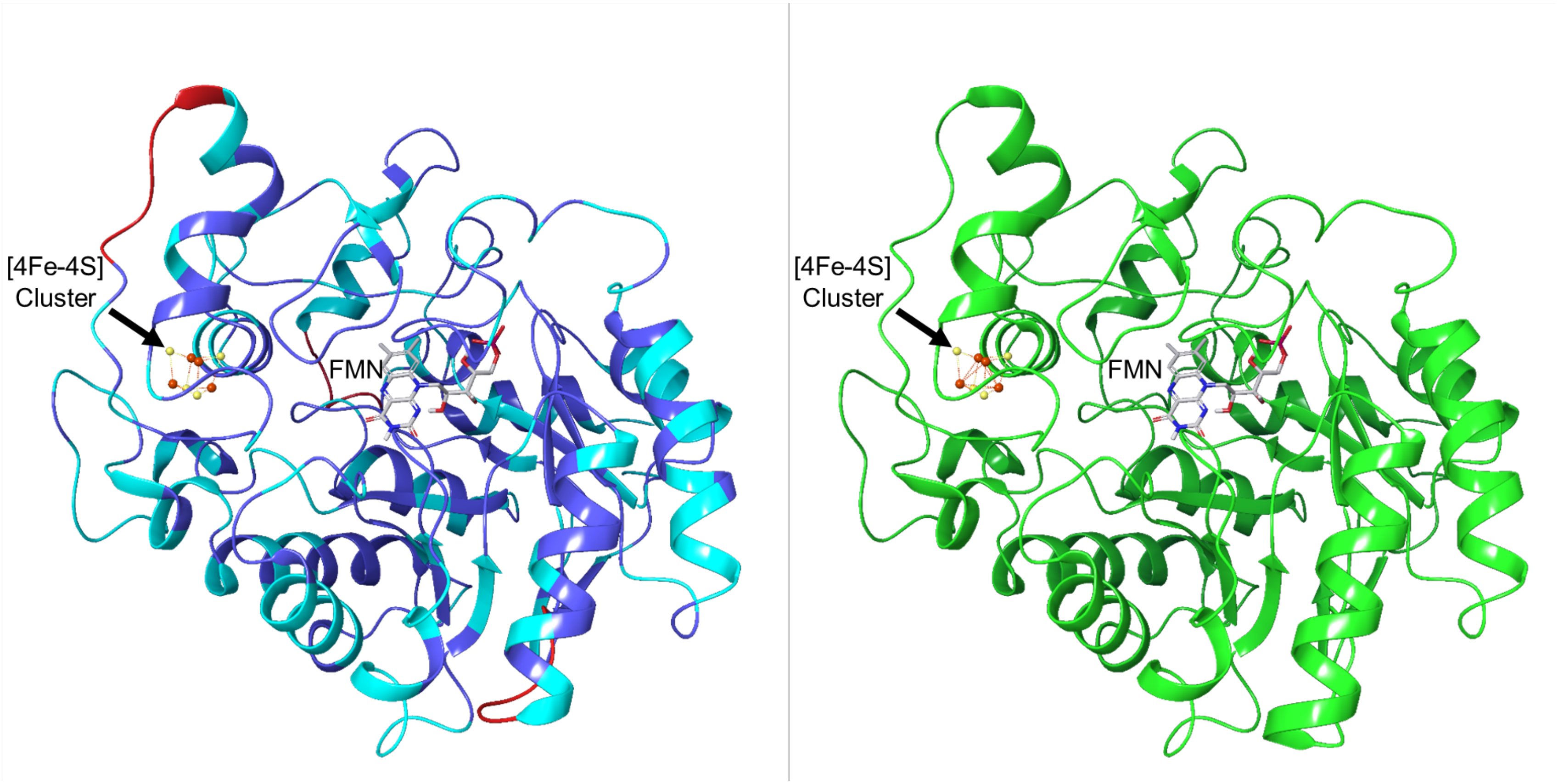

Figure S5. Structural homology of C4N14_08920 to *H. pylori* FabX. On the left is the predicted structure of C4N14_08920, colored by structure reliability. Dark blue indicates high reliability and identical residues, light blue sections are reliable with similar residues, and the red region indicates dissimilarity and uncertainty. On the right is *H. pylori* FabX (PDB ID 7E1S). The FMN and iron-sulfur cluster are shown in both.

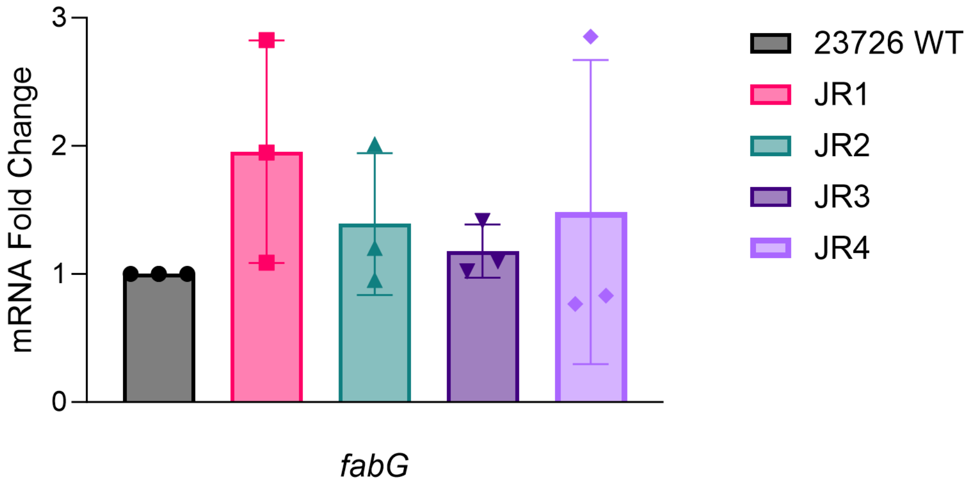

Figure S6. RT-qPCR on *fabG*. Data was normalized to 16S rRNA and fold-change is relative to the wild-type strain. JR1 and JR3 possess a C>T mutation 1bp downstream of the *fabK* transcription start site and JR1 also possesses a Gly96Ser mutation within FabK. JR2 and JR4 possess mutations upstream of *fabX* and JR4 possesses an Ala132Thr mutation in FabK. The full list of mutations can be found in **Table S1**. Shown is the mean and standard deviation of three biological replicates with each having two technical replicates. Two-way ANOVA comparing these to the wild-type showed no significant difference in any of the mutants ($p > 0.9$). Statistical analysis was performed in GraphPad Prism version 10.1.1. These results were obtained and analyzed alongside the corresponding data presented in **Figure 4A**.

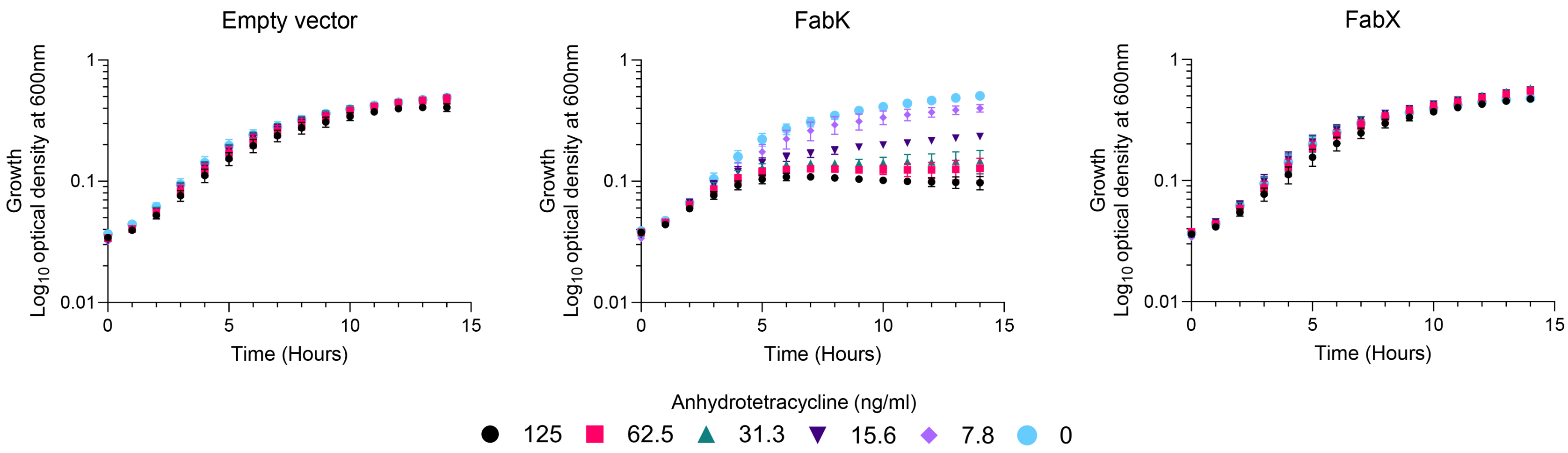

Figure S7. Growth rates of *F. nucleatum* 23726 when overexpressing FabK or FabX. The growth media Columbia broth was supplemented with a gradient of anhydrotetracycline concentrations (7.8-125 ng/ml) to induce overexpression of either FabK or FabX. Interestingly, FabK overexpression in *F. nucleatum* caused a concentration-dependent decrease in the growth; this finding is similar to previous work by Dong and Cronan[6] that ENR overexpression in *E. faecalis* was inhibited or slowed growth due to substrate competition resulting in an inhibition of unsaturated fatty acid synthesis. Shown is the mean and SEM of four biological replicates.

SI References

(1) Wu, C.; Al Mamun, A. A. M.; Luong, T. T.; Hu, B.; Gu, J.; Lee, J. H.; D'Amore, M.; Das, A.; Ton-That, H. Forward genetic dissection of biofilm development by *Fusobacterium nucleatum*: novel functions of cell division proteins FtsX and EnvC. *mBio* **2018**, *9* (2), e00360-00318. DOI: 10.1128/mBio.00360-18

(2) Zhu, L.; Bi, H.; Ma, J.; Hu, Z.; Zhang, W.; Cronan, J. E.; Wang, H. The two functional enoyl-acyl carrier protein reductases of *Enterococcus faecalis* do not mediate triclosan resistance. *mBio* **2013**, *4* (5), 10.1128/mbio.00613-00613. DOI: 10.1128/mbio.00613-13

(3) Nariya, H.; Miyata, S.; Suzuki, M.; Tamai, E.; Okabe, A. Development and application of a method for counterselectable in-frame deletion in *Clostridium perfringens*. *Applied and Environmental Microbiology* **2011**, *77* (4), 1375-1382. DOI: 10.1128/AEM.01572-10

(4) Marreddy, R. K. R.; Wu, X.; Sapkota, M.; Prior, A. M.; Jones, J. A.; Sun, D.; Hevener, K. E.; Hurdle, J. G. The fatty acid synthesis protein enoyl-ACP reductase II (FabK) is a target for narrow-spectrum antibacterials for *Clostridium difficile* infection. *ACS Infectious Diseases* **2019**, *5* (2), 208-217. DOI: 10.1021/acsinfecdis.8b00205

(5) Norseeda, K.; Bin Aziz Pavel, F.; Rutherford, J. T.; Meer, H. N.; Dureja, C.; Hurdle, J. G.; Hevener, K. E.; Sun, D. Synthesis and evaluation of phenylimidazole FabK inhibitors as new Anti-*C. Difficile* agents. *Bioorganic & Medicinal Chemistry* **2023**, *88-89*, 117330. DOI: 10.1016/j.bmc.2023.117330

(6) Dong, H.; Cronan, J. E. Unsaturated fatty acid synthesis in *Enterococcus faecalis* requires a specific enoyl-ACP reductase. *Molecular Microbiology* **2022**, *118* (5), 541-551. DOI: 10.1111/mmi.14981
